# Contrasting associations of body mass index and waist circumference with cancer incidence in the elderly: a nationwide population-based study

**DOI:** 10.3389/fonc.2025.1606686

**Published:** 2025-09-25

**Authors:** Soo Yeon Jang, Minwoong Kang, Eyun Song, Ahreum Jang, Min Jeong Park, Kyung Mook Choi, Sei Hyun Baik, Hye Jin Yoo

**Affiliations:** ^1^ Division of Endocrinology and Metabolism, Department of Internal Medicine, Korea University College of Medicine, Seoul, Republic of Korea; ^2^ Department of Biomedical Research Center, Korea University Guro Hospital, Seoul, Republic of Korea

**Keywords:** body mass index, waist circumference, cancer risk, Asians, older adults

## Abstract

**Aim:**

Obesity has been reported to be associated with increased cancer risk. Body mass index (BMI) and waist circumference (WC) are representative measures of adiposity, but BMI does not accurately reflect body composition. We aimed to evaluate the association between BMI, WC, and cancer risk in Korean old people.

**Methods:**

We used Korean National Health Insurance Service data of the elderly population (65–80 years) who underwent a health examination in 2009 without a history of malignancy. The relative hazard ratios (HRs) for cancer of the 247,625 elderly subjects were analyzed according to their BMI and WC categories.

**Results:**

During a median follow-up duration of 11.296 years, 43,369 cancer cases developed. In the fully adjusted model, the HRs for cancer incidence were significantly lower in the higher quartiles of BMI (Q1, 1; Q2, 0.920 [0.894-0.946]; Q3, 0.901 [0.873-0.930]; Q4, 0.880 [0.846-0.914], *p* < 0.001). Meanwhile, there was a sequential increase of cancer risk in the higher quartiles of WC (Q1, 1; Q2, 1.038 [1.008-1.070]; Q3, 1.074 [1.041-1.108]; Q4, 1.146 [1.103-1.190], *p* < 0.001). Even in those of normal BMI, higher WC significantly increased the cancer risk. The association between a one-standard-deviation (SD) increase in WC and cancer risk was more prominent in elderly men and the subjects with impaired fasting glucose (*p* for interaction < 0.05).

**Conclusions:**

In old individuals, increased BMI was significantly associated with a reduced cancer risk, whereas higher WC significantly increased it. WC, rather than BMI, should be applied as an anthropometric indicator in cancer prediction in older adults.

## 1 Introduction

Cancer is one of the leading causes of death worldwide, with approximately one in six people dying from cancer (1). In particular, old people aged over 60 years account for about 64% of all cancer cases and more than 70% of cancer deaths (2). Considering the recent rapid demographic shift toward an aging society, it is crucial to gain a greater insight into the risk factors associated with cancer, particularly in the elderly, for the development of effective prevention strategies.

Obesity increases the risk of certain types of cancers (3, 4), because excessive fat tissue secretes various pro-inflammatory cytokines, leading to an inflammatory environment, oxidative stress, and insulin resistance (5), which are possible causative factors for carcinogenesis (6). Although previous research has explored the positive association between body mass index (BMI), a representative indicator for general obesity, and multiple cancer types (4, 7), BMI is not a perfect measure of adiposity due to its inability to discriminate between fat and lean body mass (8). Moreover, aging is characterized by a loss of lean body mass and an increase in adipose tissue without weight gain (9). Fat and muscle are not independent of each other, exhibiting a reciprocal negative cycle between the accumulation of visceral fat and muscle atrophy (10). As a result, the association between high BMI and adverse clinical outcomes attenuates with aging (11), and this weakened relationship may be also influenced by reverse causation and increased fragility in older adults. Instead, waist circumference (WC), a more precise indicator for abdominal fat, can overcome the limitations of using only BMI as an adiposity measure, especially for the elderly. A recent meta-analysis of 69 studies involving more than 30 million participants showed that the incidence of primary liver cancer was higher in subjects with central adiposity than those with general adiposity (52.89 per 100,000 person-years vs. 39.01 per 100,000 person-years); a higher value of WC, independent of BMI, was strongly associated with the risk of cancer incidence, suggesting that central adiposity seems to contribute more to the development of cancer than general adiposity (12). With increasing age, men lose greater skeletal muscle mass and accumulate more central fat than women (13). Therefore, the association of obesity indicators such as BMI and WC with the incidence of cancer might vary according to aging and sex. However, there have been no studies on the differential relationship of BMI and WC with cancer risk by sex in an older population.

In this study, we tried to clarify the associations among BMI, WC, and cancer incidence in elderly men and women using the large-scale National Health Screening Examination (NHSE) database, adjusting for various covariates including smoking, alcohol history, physical activity, and Charlson comorbidity index (CCI), as well as mutual adjusting each general and central obesity indicator of BMI or WC.

## 2 Methods

### 2.1 Data source

This cohort study utilized Korean National Health Insurance Service (NHIS) data between 2009 and 2020. The NHIS is the single insurer that enrolls about 97% of the entire population in South Korea, and the data provided by the NHIS are widely used for nationwide population-based studies due to their representativeness of the whole Korean population (14). The NHIS database consists of insurance eligibility data including socioeconomic variables, medical history database including claims by healthcare service providers and their information, and health check-up data including questionnaires on health behavior and laboratory measurements (15). This data source was described in detail in previous studies (16, 17).

### 2.2 Study design and study participants

We included adult individuals aged between 65 and 80 years who underwent a health examination in 2009. At cohort establishment, participants who were diagnosed with cancer before baseline were excluded. We also excluded participants with an extreme BMI (<15 kg/m^2^ or ≥ 40kg/m^2^) or missing BMI data, outlier (≤ 32cm or > 129cm) or missing WC data, and those who died on the health check-up date. Ultimately, a total of 247,625 participants were enrolled in this study (**Supplementary Figure 1**). This study was approved by the Institutional Review Board of Korea University (IRB no. 2024GR0238), and the NHIS review committee granted us permission to use the NHIS data (NHIS-2022-1-704). The need for informed consent was waived because anonymous and de-identified data were used for the study.

### 2.3 Study outcomes

The primary study outcome was the incidence of cancer. Cancer was defined as the presence of more than 3 claims in a year for the same C-code based on International Classification of Diseases 10^th^ Revision (ICD-10), or more than 1 claim for admission within a year under any C-code. The NHIS meticulously reviews C-codes due to their potential advantages to patients (18). Study participants were followed from the date of the baseline health examination until the incident cancer, death, or the end of the study (2020.12.31), whichever came first.

### 2.4 Exposure

BMI was calculated as measured body weight (kg) divided by measured height squared (m^2^). WC was measured at the narrowest point between the lowest part of the rib and the iliac crest under comfortable breathing. The measurements were conducted by trained staff as the standardized health examinations. In order to evaluate the tendency of cancer risk according to BMI and WC, adiposity markers were divided into quartiles. Furthermore, BMI was categorized based on clinically significant cutoffs to validate whether the relationship between WC and cancer risk was maintained within each BMI range.

### 2.5 Covariates

Data for age, sex, and income were obtained from the insurance eligibility database of the NHIS. Data on health-related behaviors, including smoking, drinking, and exercise habits, were obtained from the health examination questionnaire database. Smoking status was categorized as never-smoker, ex-smoker, current-smoker, or unknown. Alcohol consumption was classified as none, moderate (1–14 cups per week in men, 1–7 cups per week in women), heavy (≥ 15 cups per week in men, ≥ 8 cups per week in women) (19), or unknown. Exercise habits were divided into none, regular (high intensity physical activity ≥ 3 days/week or moderate intensity physical activity ≥ 5 days/week) (20), irregular (other physical activities), or unknown based on questionnaires on frequency and intensity of physical activity. Social income was classified into 4 groups according to percentile of insurance premiums (Medicaid/1-30/31-70/> 70) (21). CCI was scored to adjust for comorbidities based on claim codes during the year prior to the health screening; different scores based on one-year mortality risk are designated for each disease, and the sum of the weighted scores are given to individuals with more than one comorbid disease (22).

### 2.6 Other variables of baseline characteristics

Systolic and diastolic blood pressure, fasting blood sugar (FBS), total cholesterol, high-density lipoprotein cholesterol (HDL-C), low-density lipoprotein cholesterol (LDL-C), triglyceride, aspartate aminotransferase (AST), alanine aminotransferase (ALT), and creatinine levels were obtained from the health examination database. Blood samples were extracted after at least 8 hours of fasting.

### 2.7 Statistical analysis

The baseline characteristics are presented as mean ± standard deviation (SD) for continuous variables and numbers (%) for categorical variables. Independent t-tests for continuous variables and chi-square tests for categorical variables were performed to compare values between sexes. Study subjects were categorized into 4 groups according to the quartile of BMI and WC in total, and according to sex. Comparisons according to quartile groups were performed using ANOVA and chi-square tests.

Cox proportional hazards model was used to calculate hazard ratios (HRs) with 95% confidence intervals (CIs) for incident cancer. To minimize effects of confounding factors, we adjusted for variables that can affect the study outcome. In Model 1, we adjusted for age and sex at baseline. In Model 2, we further adjusted for alcohol, smoking, exercise, FBS, income, and CCI. In Model 3, WC (BMI) was further adjusted for. To evaluate the independent effects of BMI and WC on cancer risk, as indicators of general and central adiposity respectively, we conducted mutual adjustment of these variables. We also divided men and women and BMI (WC) deciles, and we calculated HRs and 95% CIs for developing 24 specific types of cancer (oral, esophagus, stomach, colorectal, liver, biliary, pancreas, laryngeal, lung, breast, cervical, corpus, ovary, prostate, testicular, renal, bladder, central nervous system, thyroid, Hodgkin, non-Hodgkin, multiple myeloma, leukemia, etc). Furthermore, we categorized subjects by clinically significant BMI cutoff values based on the Korean Society for the Study of Obesity (KSSO) guideline (23) and calculated the risk of cancer development according to WC quartiles in each BMI category. Then, stratified analysis and interaction testing were conducted using likelihood ratios to assess the potential effect modification by sex, smoking, alcohol, exercise, income, CCI, and FBS. In these subgroup analyses, HRs (95% CIs) for cancer incidence were compared by a 1-SD increase in BMI or WC. Sensitivity analysis excluding cancer cases diagnosed within two years from the baseline was also conducted to preclude possible effects on body weight caused by cancer. We examined the proportional hazards assumption using plots of the log(−log) survival function and Schoenfeld residuals. *P* < 0.05 was considered statistically significant. All analyses were performed using Statistical Analysis System (RRID: SCR_008567) version 9.4 (SAS Institute Inc., Cary, NC, USA).

## 3 Results

### 3.1 Baseline characteristics of study participants

The baseline characteristics of the study population are presented in **Table 1**. Among 247,625 participants, 126,335 were men and 121,290 were women (mean age, 70.0 years). Participants in higher BMI quartile groups had higher WC, blood pressure, FBS, total cholesterol, LDL-C, triglyceride, AST, and ALT values (**Supplementary Table 1-1**). This trend persisted when categorizing the study population according to WC quartiles (**Supplementary Table 1-2**).

**Table 1:** Baseline characteristics of study participants.

	Total (N = 247625)	Men (N = 126335)	Women (N = 121290)	P-value
Age (years)	70.0 ± 3.7	69.8 ± 3.6	70.3 ± 3.8	<0.001
BMI (kg/m^2^)	23.5 ± 3	23.2 ± 2.8	23.9 ± 3.2	<0.001
WC (cm)	82.0 ± 8.2	83.4 ± 7.9	80.6 ± 8.2	<0.001
Systolic BP (mmHg)	130.6 ± 16.8	131.0 ± 16.8	130.2 ± 16.7	<0.001
Diastolic BP (mmHg)	78.9 ± 10.3	79.3 ± 10.4	78.4 ± 10.3	<0.001
Fasting glucose (mg/dL)	98.1 ± 19.9	99.4 ± 21.5	96.7 ± 18.0	<0.001
Total cholesterol (mg/dL)	200.4 ± 44.8	192.5 ± 44.6	208.7 ± 43.6	<0.001
HDL-C (mg/dL)	55.8 ± 37.5	54.9 ± 35.9	56.9 ± 38.9	<0.001
LDL-C (mg/dL)	121.5 ± 71.7	114.7 ± 67.0	128.6 ± 75.6	<0.001
Triglyceride (mg/dL)	135.7 ± 81.4	133.9 ± 86	137.6 ± 76.4	<0.001
AST (U/L)	26.1 ± 16.9	27.2 ± 17.3	24.9 ± 16.3	<0.001
ALT (U/L)	21.7 ± 17.3	23.2 ± 17.5	20.2 ± 16.9	<0.001
Creatinine (mg/dL)	1.0 ± 1.1	1.1 ± 1.2	0.9 ± 1.0	<0.001
eGFR (mL/min/1.73m^2^)	61 ± 19.1	63.4 ± 19.1	58.5 ± 18.8	<0.001
Smoking status [n (%)]				<0.001
Never	169131 (68.3)	52895 (41.9)	116236 (95.8)	
Ex-smoker	35718 (14.4)	34534 (27.3)	1184 (1)	
Current	41105 (16.6)	38148 (30.2)	2957 (2.4)	
Unknown	1671 (0.7)	758 (0.6)	913 (0.8)	
Drinking habit [n (%)]				<0.001
None	168949 (68.2)	60211 (47.7)	108738 (89.7)	
Moderate	46821 (18.9)	39146 (31)	7675 (6.3)	
Heavy	25967 (10.5)	24045 (19)	1922 (1.6)	
Unknown	5888 (2.4)	2933 (2.3)	2955 (2.4)	
Exercise [n (%)]				<0.001
None	78049 (31.5)	34889 (27.6)	43160 (35.6)	
Irregular	118387 (47.8)	58965 (46.7)	59422 (49)	
Regular	49634 (20)	31673 (25.1)	17961 (14.8)	
Unknown	1555 (0.6)	808 (0.6)	747 (0.6)	
CCI [n (%)]				<0.001
0	167815 (67.8)	88498 (70.1)	79317 (65.4)	
1	63167 (25.5)	30625 (24.2)	32542 (26.8)	
2	14012 (5.7)	6086 (4.8)	7926 (6.5)	
≥3	2631 (1.1)	1126 (0.9)	1505 (1.2)	
Income [n (%)]				<0.001
Medicaid	1298 (0.5)	505 (0.3)	793 (0.7)	
1-30%	60811 (24.6)	35390 (28.0)	25421 (21.0)	
31-70%	73594 (29.7)	37979 (30.1)	35615 (29.3)	
>70%	109573 (44.2)	51400 (40.7)	58173 (48.0)	
Unknown	2349 (0.9)	1061 (0.8)	1288 (1.1)	

Values are presented as number (%), mean ± standard deviation.

BP, blood pressure; HDL-C, high-density lipoprotein cholesterol; LDL-C, low-density lipoprotein cholesterol; AST, aspartate aminotransferase; ALT, alanine aminotransferase; eGFR, estimated glomerular filtration rate; CCI, Charlson comorbidity index.

### 3.2 Risk of total and specific cancer according to BMI category

**Figure 1:**
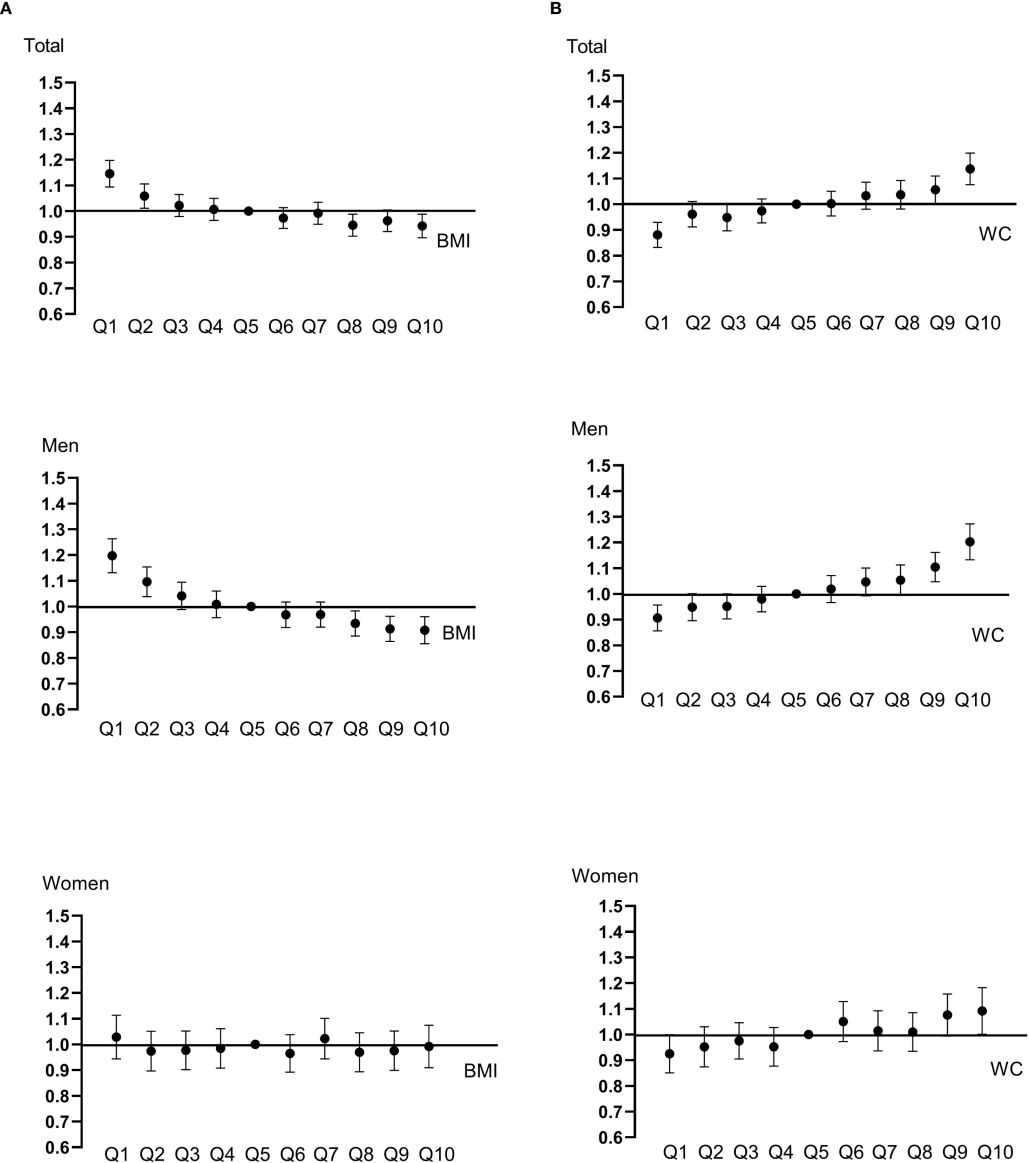
Hazard ratios and confidence intervals for developing cancer were calculated with Cox proportional hazard models. BMI **(A)** and WC **(B)** were categorized into deciles. Models were adjusted for age, sex, alcohol, smoking, exercise, fasting blood glucose, income, Charlson comorbidity index, and mutually adjusted for WC and BMI.

During the median follow-up duration of 11.296 years, 43,369 cancer cases developed. The HRs with 95% CIs of overall cancer risk according to BMI and WC quartiles are shown in **Table 2**. When categorizing the participants according to BMI quartile, there was a sequential decrease in overall cancer risk in the higher quartiles (**Table 2-1**). In the fully adjusted model, the highest BMI quartile group showed a 12% decrease in cancer risk compared to the lowest quartile group (Model 3, Q1, reference; Q2, 0.920 [0.894-0.946]; Q3, 0.901 [0.873-0.930]; Q4, 0.880 [0.846-0.914], *p* < 0.001). Furthermore, when BMI increased by 1SD, 5.4% of cancer risk significantly decreased. The gradual decline in cancer risk was also observed when subjects were divided into decile groups according to BMI; in men, a consistent pattern was observed with statistical significance, whereas the trend did not persist in women (**Figure 1**, **Supplementary Table 2-1**). In particular, the risk of esophagus, stomach, colorectal, lung cancer showed a significantly sequential decrease in cancer risk across BMI quartiles (**Supplementary Figure 2-1**, **Supplementary Table 3-1**).

**Table 2-1:** Hazard ratios and confidence intervals for developing cancer according to quartiles of BMI.

BMI	Range	Events (n)	Follow-up duration (person-year)	Incidence rate (per 1000 person-years)	Crude	Model 1	Model 2	Model 3
Total								
1SD					0.925 (0.916 - 0.934)	0.981 (0.971 - 0.990)	0.998 (0.988 - 1.008)	0.946 (0.931 - 0.960)
Q1 (n=60,665)	15.0-21.4	11344	578540.2382	19.60797063	1	1	1	1
Q2 (n=62,492)	21.5-23.4	11050	621734.2396	17.77286708	0.905 (0.882 - 0.929)	0.937 (0.913 - 0.962)	0.962 (0.937 - 0.988)	0.920 (0.894 - 0.946)
Q3 (n=61,546)	23.5-25.4	10686	620301.4757	17.22710717	0.877 (0.854 - 0.900)	0.934 (0.909 - 0.959)	0.972 (0.946 - 0.998)	0.901 (0.873 - 0.930)
Q4 (n=62,922)	25.5-39.8	10289	640508.5859	16.06379716	0.817 (0.796 - 0.840)	0.945 (0.920 - 0.971)	0.989 (0.962 - 1.017)	0.880 (0.846 - 0.914)
p-value					<0.001	<0.001	0.019	<0.001
p-for trend					<0.001	<0.001	0.565	<0.001
Men								
1SD					0.943 (0.932 - 0.954)	0.957 (0.947 - 0.968)	0.979 (0.968 - 0.991)	0.918 (0.901 - 0.935)
Q1 (n=31,937)	15.0-21.2	7892	285851.1129	27.60877829	1	1	1	1
Q2 (n=31,312)	21.3-23.1	7370	295381.1964	24.95080963	0.901 (0.873 - 0.931)	0.919 (0.89 - 0.948)	0.947 (0.918 - 0.978)	0.900 (0.869 - 0.931)
Q3 (n=31,392)	23.2-25.0	7286	301515.5866	24.16458825	0.872 (0.845 - 0.901)	0.9 (0.872 - 0.929)	0.946 (0.916 - 0.977)	0.867 (0.834 - 0.901)
Q4 (n=31,694)	25.1-39.8	7248	306416.7858	23.65405662	0.854 (0.827 - 0.881)	0.888 (0.86 - 0.917)	0.943 (0.913 - 0.974)	0.824 (0.786 - 0.863)
p-value					<0.001	<0.001	0.001	<0.001
p-for trend					<0.001	<0.001	0.001	<0.001
Women								
1SD					1.018 (1.001 - 1.035)	1.030 (1.012 - 1.047)	1.031 (1.013 - 1.048)	1.031 (1.013 - 1.049)
Q1 (n=29,835)	15.0-21.7	3209	307230.9076	10.44491267	1	1	1	1
Q2 (n=30,230)	21.8-23.7	3370	318476.668	10.58162289	1.012 (0.964 - 1.062)	1.036 (0.987 - 1.087)	1.040 (0.991 - 1.092)	1.009 (0.959 - 1.063)
Q3 (n=31,173)	23.8-25.9	3489	329191.4689	10.59869508	1.013 (0.966 - 1.063)	1.044 (0.995 - 1.096)	1.049 (1.000 - 1.101)	0.997 (0.943 - 1.055)
Q4 (n=30,052)	26.0-39.8	3505	317020.8131	11.05605643	1.058 (1.009 - 1.110)	1.091 (1.040 - 1.145)	1.094 (1.042 - 1.148)	1.008 (0.943 - 1.077)
p-value					0.099	0.005	0.004	0.953
p-for trend					0.028	0.001	<0.001	0.941

BMI, body mass index; SD, standard deviation.

Model 1: adjusted for age, sex.

Model 2: adjusted for model 1 plus alcohol, smoking, exercise, fasting blood glucose, income, Charlson comorbidity index.

Model 3: adjusted for model 2 plus WC.

In sex-stratified analyses, sex was excluded from the covariates.

### 3.3 Risk of total and specific cancer according to WC category

When categorizing participants according to WC quartile, there was a sequential increase in overall cancer risk in the higher quartiles (**Table 2-2**). This sequential increase persisted in all adjusted models, maintaining statistical significance (*p* < 0.001). Participants in the highest WC quartile exhibited a 14.6% increase in the risk of developing cancer compared with the lowest quartile group (Model 3, Q1, reference; Q2, 1.038 [1.008-1.070]; Q3, 1.074 [1.041-1.108]; Q4, 1.146 [1.103-1.190], *p* < 0.001). Moreover, with every 1-SD increase in WC, the risk of cancer increased by 7.2%. When the subjects were divided into deciles of WC, a continuously rising trend in cancer risk was also observed; an increasing tendency of cancer risk with increasing WC was noted in both men and women, but was more prominent in men (**Figure 1**, **Supplementary Table 2-2**). In particular, the risk of esophagus, colorectal, lung cancer exhibited significant sequential increases across WC quartiles (**Supplementary Figure 2-2**, **Supplementary Table 3-2**).

**Table 2-2:** Hazard ratios and confidence intervals for developing cancer according to quartiles of WC.

WC	Range	Events (n)	Follow-up duration (person-year)	Incidence rate (per 1000 person-years)	Crude	Model 1	Model 2	Model 3
Total								
1SD					1.089 (1.079 - 1.099)	1.018 (1.008 - 1.028)	1.027 (1.017 - 1.037)	1.072 (1.056 - 1.088)
Q1 (n=62,703)	33-76	9818	621997.139	15.78463852	1	1	1	1
Q2 (n=55,630)	77-81	9432	556759.269	16.94089443	1.073 (1.043 - 1.104)	1.001 (0.973 - 1.030)	1.012 (0.984 - 1.041)	1.038 (1.008 - 1.070)
Q3 (n=68,186)	82-87	12264	679358.371	18.05232779	1.143 (1.114 - 1.174)	1.006 (0.979 - 1.033)	1.027 (1.000 - 1.055)	1.074 (1.041 - 1.108)
Q4 (n=61,106)	88-129	11855	602969.7604	19.66101914	1.246 (1.213 - 1.280)	1.039 (1.012 - 1.068)	1.065 (1.036 - 1.094)	1.146 (1.103 - 1.190)
p-value					<0.001	0.011	<0.001	<0.001
p-for trend					<0.001	0.005	<0.001	<0.001
Men								
1SD					0.998 (0.987 - 1.010)	1.005 (0.994 - 1.017)	1.017 (1.005 - 1.029)	1.087 (1.067 - 1.107)
Q1 (n=33,186)	33-78	7776	304735.1102	25.51724347	1	1	1	1
Q2 (n=29,988)	79-83	7017	283715.896	24.73248803	0.968 (0.937 - 0.999)	0.984 (0.953 - 1.017)	1.003 (0.971 - 1.036)	1.052 (1.016 - 1.089)
Q3 (n=30,766)	84-88	7226	293294.4805	24.6373542	0.964 (0.933 - 0.995)	0.984 (0.953 - 1.017)	1.017 (0.984 - 1.050)	1.100 (1.059 - 1.143)
Q4 (n=32,395)	89-129	7777	307419.1951	25.29770465	0.990 (0.959 - 1.021)	1.008 (0.977 - 1.040)	1.038 (1.006 - 1.072)	1.179 (1.126 - 1.234)
p-value					0.07	0.372	0.091	<0.001
p-for trend					0.49	0.64	0.015	<0.001
Women								
1SD					1.047 (1.029 - 1.064)	1.045 (1.028 - 1.063)	1.044 (1.027 - 1.062)	1.049 (1.023 - 1.076)
Q1 (n=31,939)	36-75	3365	332785.4374	10.11162035	1	1	1	1
Q2 (n=29,185)	76-80	3196	307670.2396	10.38774502	1.027 (0.979 - 1.078)	1.033 (0.984 - 1.084)	1.033 (0.985 - 1.085)	1.030 (0.979 - 1.084)
Q3 (n=27,549)	81-85	3170	289716.0164	10.94174923	1.082 (1.031 - 1.136)	1.089 (1.037 - 1.143)	1.091 (1.039 - 1.145)	1.085 (1.027 - 1.147)
Q4 (n=32,617)	86-129	3842	341748.1643	11.24219645	1.112 (1.061 - 1.164)	1.109 (1.059 - 1.161)	1.106 (1.056 - 1.159)	1.097 (1.030 - 1.169)
p-value					<0.001	<0.001	<0.001	0.011
p-for trend					<0.001	<0.001	<0.001	0.001

WC, waist circumference; SD, standard deviation.

Model 1: adjusted for age, sex.

Model 2: adjusted for model 1 plus alcohol, smoking, exercise, fasting blood glucose, income, Charlson comorbidity index.

Model 3: adjusted for model 2 plus BMI.

In sex-stratified analyses, sex was excluded from the covariates.

### 3.4 Risk of cancer according to WC by BMI category

**Figure 2:**
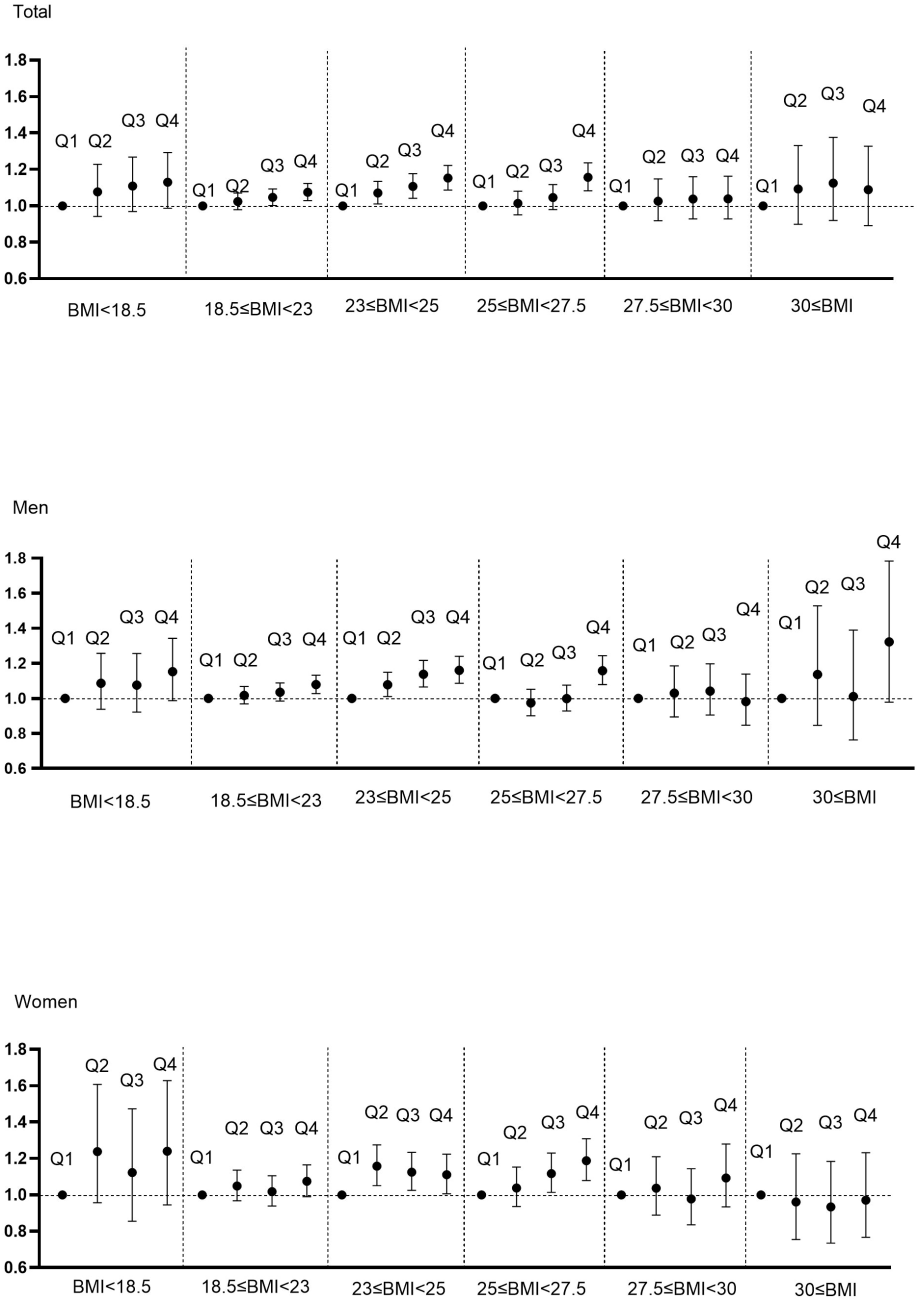
Hazard ratios for cancer incidence using Cox proportional hazard model according to WC quartile and BMI category. BMI was categorized into BMI<18.5, 18.5≤BMI<23, 23≤BMI<25, 25≤BMI<27.5, 27.5≤BMI<30, 30≤BMI according to classification criteria for Asians. Adjusted for age, sex, alcohol, smoking, exercise, fasting glucose, income, Charlson comorbidity index.

We evaluated whether the association between WC and cancer risk is maintained in respective BMI categories (**Figure 2**, **Supplementary Figure 3**, **Supplementary Table 4**). Even in the same BMI range, those with higher WC quartiles showed a trend in increased risk of developing cancer. Notably, those in the normal range of BMI (18.5 ≤ BMI < 23) also exhibited an increasing tendency of cancer risk with higher WC quartiles, but this phenomenon was statistically significant only in the elderly men, and not in women.

### 3.5 Subgroup analysis

**Figure 3:**
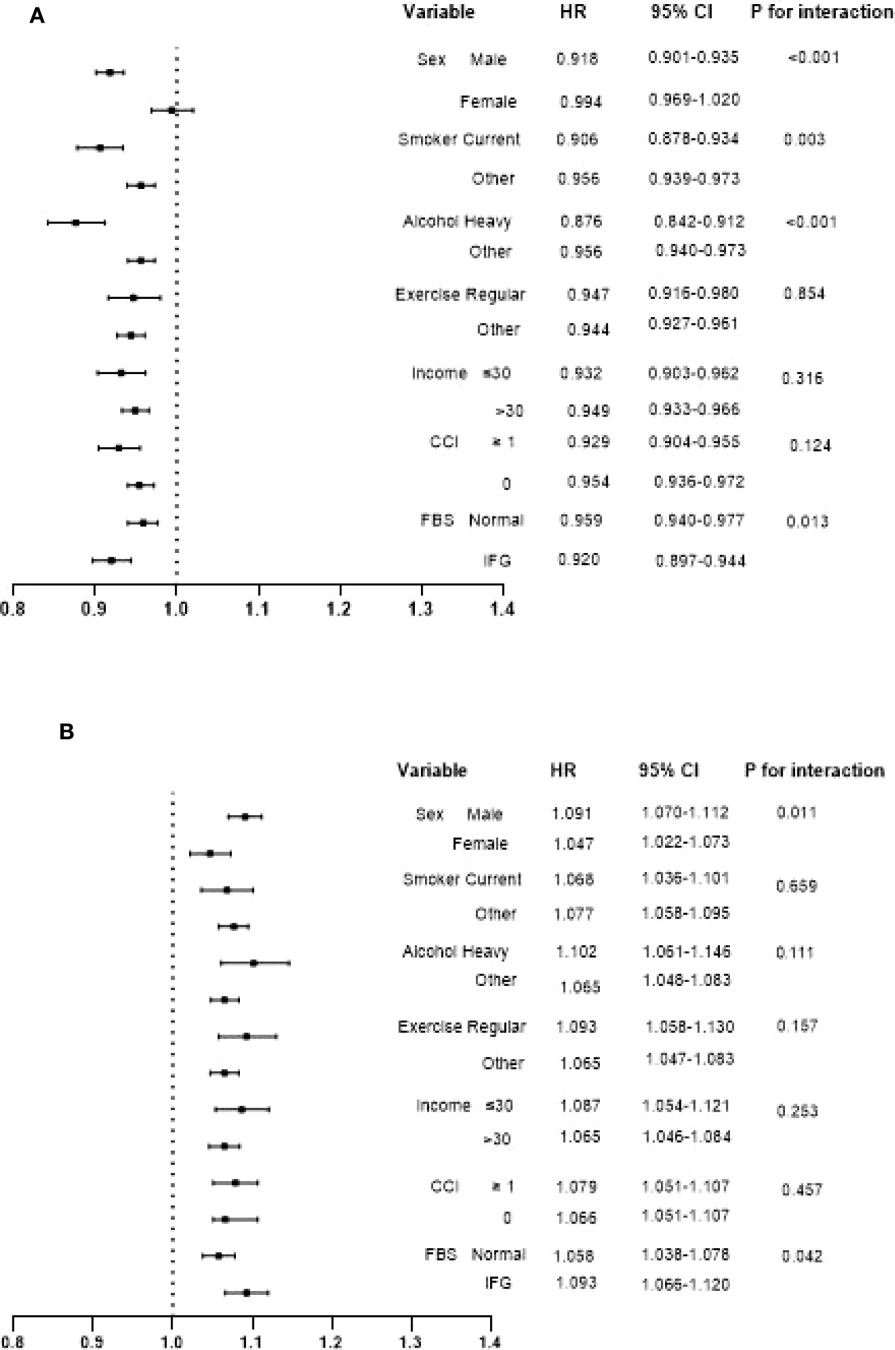
Interaction of hazard ratios and confidence intervals for cancer incidence per 1-SD increase in BMI **(A)** and WC **(B)** in each subgroup. Adjusted forage, sex, alcohol, smoking, exercise, fasting blood glucose, income, Charlson comorbidity index, and mutually adjusted for WC and BMI.

Stratification was performed according to sex, smoking, alcohol, exercise, income, CCI, and FBS, and cancer risk per 1-SD increase in BMI (or WC) was analyzed respectively (**Figure 3**). A contrasting association of BMI and WC with cancer risk was observed in all subgroups. However, this negative association between a 1-SD increase in BMI and cancer incidence was more prominent in men, current smokers, heavy alcoholics and individuals with impaired fasting glucose (IFG). Meanwhile, the effect of a 1-SD increase of WC on the higher risk of cancer was also more obvious in men and subjects with IFG.

### 3.6 Sensitivity analysis

As cancer development can significantly affect body weight and diagnosis is often not simultaneous with the onset of cancer development, we conducted sensitivity analysis after excluding patients who were diagnosed with cancer within two years from baseline health examination. Results were consistent with the main analysis (**Supplementary Table 5**).

## 4 Discussion

In this study, we showed the different association of BMI and WC with cancer incidence in the Korean elderly population. Increased BMI was associated with decreased cancer risk, which was more prominent in men, current smokers, heavy drinkers, and those with IFG. In contrast, higher WC was associated with increased cancer risk, which was more apparent in men and subjects with IFG. A tendency toward increased cancer risk according to the higher WC was maintained in each BMI range, even in the normal BMI category.

Obesity was related to high malignancy risk in previous studies. Increased adipose tissue promotes fatty acid synthase and produces high levels of free fatty acids, which increase sensitivity to oncogenic signals (24). Enhanced estrogen production and changes in sex hormone metabolism due to aromatization in adipose tissue have also been suggested as an explanation for the link between obesity and cancer (25). Mesenteric fat, which contributes to abdominal obesity, is especially critical because it poses higher lipogenicity and is metabolically active compared to fat in other parts (24). Even though body fat distribution more importantly mediates metabolic disturbance than general obesity, BMI has been widely used as a practical measure for adiposity due to its simplicity and standardization, despite being an imperfect indicator of body fat distribution. In 2018, the World Cancer Research Fund reported that high BMI was associated with higher risk for 12 cancers, including colorectal, postmenopausal breast, esophageal, pancreatic, liver, kidney, oral/pharyngeal/laryngeal, stomach cardia, gallbladder, ovarian, prostate, and uterine cancers (26). In a recent large population-based study of Swedish young men, BMI at age 18 was linearly associated with risk of developing all 18 site-specific cancers during a mean follow-up period of 31 years (27). However, all these studies have been conducted in young or middle-aged populations, not focusing on the elderly. In the present study, we for the first time documented that the relative HR for the incidence of cancer decreased by 5.4% per 1-SD increase in BMI and the group with the highest quartile of BMI exhibited a 12% reduction in cancer risk when compared to the lowest after adjusting for other covariates in older adults. These results were quite contradictory to previous research showing a positive relationship between BMI and cancer risk. BMI in the elderly population represents adiposity inaccurately; a higher BMI in older individuals can be the result of more lean body mass or more fat mass. The function of fat mass as nutritional reserves becomes more important in advanced age and measures of BMI late in life are more likely to be confounded by comorbid medical conditions (28). As a result, Jee et al. (29) reported that a relative increase in the risk of death due to high BMI was observed among subjects younger than 50 years, but not for those 65 years or older at baseline. Jacobs et al. (30) also demonstrated that BMI before age 50 is more strongly associated with pancreatic cancer risk than BMI at older ages, suggesting that early-life BMI has a lasting impact on cancer risk, but the impacts of late-life BMI may not be as clear. Our data lacked prior BMI trajectory, therefore we could not evaluate the long-term impact of weight changes or the age-specific effects of BMI. In the present study with mean age of 70.0 years, the risk of cancer decreased sequentially according to higher BMI quartile, and interestingly such negative association was found only in elderly men. Despite greater skeletal muscle mass in men than in women throughout the entire lifespan, men start to lose muscle mass at the end of their fifth decade at a rapid rate (31); thus, the physiologic function of muscle for healthy aging would be more critical in men than women. Previously, we reported that both fat mass and muscle mass index were independent risk factors for the development of diabetes in men aged 70 years or older, whereas in women, fat mass was independently associated with diabetes, but not muscle (32). In addition, current smokers, heavy drinkers, and those with IFG showed a more prominent reverse correlation between BMI and cancer risk. This implies that in those with an unhealthy lifestyle or hyperglycemia, weight loss may contribute to cancer development, suggesting the importance of maintaining optimal weight in older adults with unhealthy lifestyle habits or prediabetes.

To identify the potential confounding residual effect of smoking, we conducted the analysis according to smoking history with excluding the first three years of cancer cases to minimize bias from chronic diseases (**Supplementary Table 6**). Interestingly, among never smokers, the negative relationship between BMI and cancer risk was not observed before adjusting for WC. When adjusting for WC, assuming that visceral fat content is the same, the reverse association between BMI and cancer risk became evident, especially in men. Higher BMI would imply more metabolically healthy or less fragile state with higher muscle mass, leading to decreased cancer risk. Among ever-smokers, who carry smoking as an inherent cancer risk factor, consistent results indicating a reverse relationship between BMI and cancer risk were observed. This suggests that smokers with low BMI may be more vulnerable to increased cancer risk.

In contrast to BMI, WC is more intimately correlated with visceral adipose tissue, the body composition component that most strongly causes insulin resistance. After adjustment for BMI, larger WC reflected higher visceral fat. There have been few reports of the effects of BMI adjustment on associations between WC and cancer risk. A nationwide population‐based cohort study showed that only WC was associated with colorectal cancer risk when WC and BMI were mutually adjusted for (33). A recent study from Japan also demonstrated no significant association between BMI and the risk of colorectal cancer (34). Katzmarzyk et al. (35) demonstrated that visceral adipose tissue remained significantly associated with cancer incidence (HR, 1.22; 95% CI, 1.03–1.46) after adjusting for total fat mass, but not vice versa. Furthermore, in the Framingham Heart Study Cohort, the risk of cancer incidence increased by 43% per 1-SD increase in visceral adipose tissue (36), and a recent review of 22 studies found negative associations between visceral adipose tissue and survival among patients with colorectal and pancreatic cancers (37). Likewise, in the present study, the risk of cancer incidence was significantly increased by 14.6% in subjects with the highest quartile of WC when compared to the lowest after adjustment for other covariates including BMI; this association was more prominent in elderly men than women (*p* for interaction=0.011). This suggests the significance of central obesity management especially in elderly men for cancer prevention. WC is an indicator of visceral fat. Visceral fat could contribute to metabolic abnormalities and accelerated proinflammatory responses, thus leading to tumorigenesis. In men, higher amounts of visceral fat might explain the prominent relationship between WC and cancer risk, although body composition data were not available in this study. Furthermore, despite the rapid decline in estrogen level after menopause, relatively higher estrogen levels or hormone sensitivity in women compared to men may have exerted some protective effects against cancer development. Postmenopausal women would be vulnerable to non-cancer deaths such as cardiovascular diseases or fracture, and this might have influenced the association between WC and cancer risk in women. Since our data lacked information on cause of death, future research accounting for competing risk is needed. Although the underlying mechanisms of these sex-based differences could not be clarified in this study, the different application based on age, sex, and ethnicity characteristics might be crucial to determine which obesity parameters most strongly predict cancer risk. Future research integrating hormonal status by sex, inflammatory markers and metabolic indicators would be necessary. This would be the basis for the personalized cancer prevention strategies for older adults. Furthermore, the IFG group demonstrated a more evident positive association between WC and cancer risk, which implies a synergistic effect of mesenteric fat and hyperglycemia on cancer development. Interestingly, in each BMI category, even in the normal range of BMI, a positive association between WC and cancer risk was observed. These results emphasized the importance of measuring WC in combination with BMI in older adults for accurate prediction of cancer risk.

Our study has some limitations. First, dynamic changes and variability of adiposity markers that might influence cancer incidence during the follow-up period were not considered. Incorporating longitudinal changes of adiposity and their associations with cancer risk would be intriguing research topic in the future. Second, exact body composition was not evaluated in this study due to lack of data. Muscle and fat could be interconnected each other physically and through mediators called myokines and adipokines. Decreased muscle mass may lead to insulin resistance, which favors fat accumulation. Fatty infiltration of muscle may disrupt metabolism and normal physiology of muscle, accelerating muscle loss. Body composition rather than body weight or BMI would appropriately explain the contributions to cancer risk. Lack of data on body composition is one of limitations of our study, necessitating future research based on specific body composition analysis. Furthermore, BMI in older adults could be affected by frailty, sarcopenia, or weight changes accompanied by various comorbidities. Although we adjusted CCI accordingly, it is likely that effects of comorbidities or preclinical disease on body weight was not fully accounted for. The interpretation of BMI results should be approached with caution, and further studies to investigate the association between body composition and cancer risk using imaging modalities, and according to respective medical conditions in the elderly are needed. Third, nonsignificant results were observed in some specific cancer types, probably due to insufficient sample size. Given the small number of rare cancer cases, the results are exploratory in nature with limited statistical power. Additionally, heterogenicity in specific cancer types, such as staging at diagnosis or pathologic findings, was not considered in this study. Future research with a larger sample size for particular cancers should explore the underlying mechanisms specific to cancer origins and pathologic types, especially in older people. Fourth, collider bias from mutual adjustment of BMI and WC is likely in the presence of unmeasured factors affecting both BMI and WC. However, we assumed that major shared determinants such as lifestyle habits were adequately controlled through including lifestyle factors as covariates. BMI and WC are partially independent markers of adiposity, each carrying different implications for metabolic and cancer risk. BMI represents overall adiposity, whereas WC more specifically reflects central obesity, which is closely associated with visceral fat. Given their distinct roles, we adjusted each measure to disentangle their independent relationships with cancer incidence, assuming that no unmeasured common causes act as colliders. Future studies considering causal relationships and temporal dynamics are warranted. Fifth, we were unable to consider non-cancer deaths as competing risks due to a lack of data on causes of death. Lastly, the timing of diagnosis might not exactly reflect the onset of cancer. To minimize bias from the impact of occult cancer on adiposity markers, we conducted sensitivity analysis excluding cancer cases diagnosed within two years from baseline and found consistent results.

The strength of our study is that it is the first longitudinal nationwide study to present contrasting associations of BMI and WC with cancer risk in elderly Asians. Compared with Caucasians, Asians have higher amounts of abdominal adipose tissue and lower muscle mass for a given BMI, leading to a greater tendency toward central obesity and higher susceptibility to metabolic disturbance-related outcomes (38). Further research including other ethnicities such as Europeans, and comparative analyses with the current findings would be useful.

## 5 Conclusion

In conclusion, as cancer prediction markers, WC would be still useful even in older people, whereas BMI requires careful interpretation when applied to older adults. Body composition analysis and its correlation with cancer risk in old individuals according to sex, as well as the possible protective effect of muscle in older men, requires further investigation. Maintaining a healthy body weight is important in older people. Even in older individuals of normal weight, central obesity should be strictly controlled for cancer prevention. Future intervention studies should explore effective control of central obesity with nutrition, medication, and exercise, especially in the elderly population. Current cancer screening is primarily based on age and does not adequately reflect individual risk factors. Even within the same age group, especially in older adults, personalized cancer screening strategies according to risk stratification would be needed. For example, older adults with central obesity despite a normal body weight, especially in men or IFG, may benefit from more rigorous cancer screening approach.

## Data Availability

The original contributions presented in the study are included in the article/**Supplementary Material**. Further inquiries can be directed to the corresponding author.
