# Supplementary figures and images for: Contrasting associations of body mass index and waist circumference with cancer incidence in the elderly: a nationwide population-based study
Source: Front Oncol. 2025 Sep 25;15:1606686. doi: 10.3389/fonc.2025.1606686 (PMC12507619; doi:10.3389/fonc.2025.1606686)

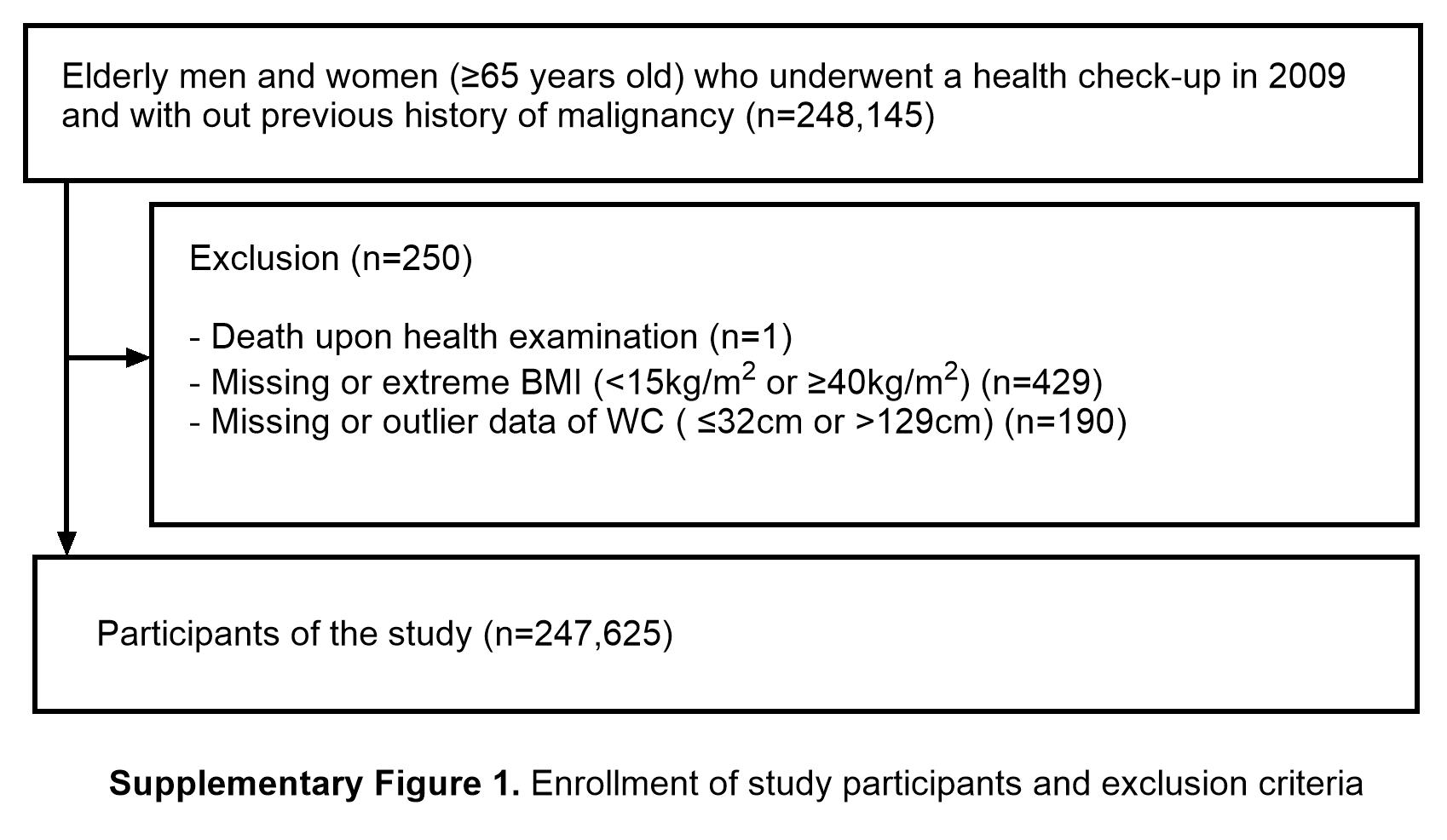

Supplement: Supplementary file 1 [file Image1.jpeg]

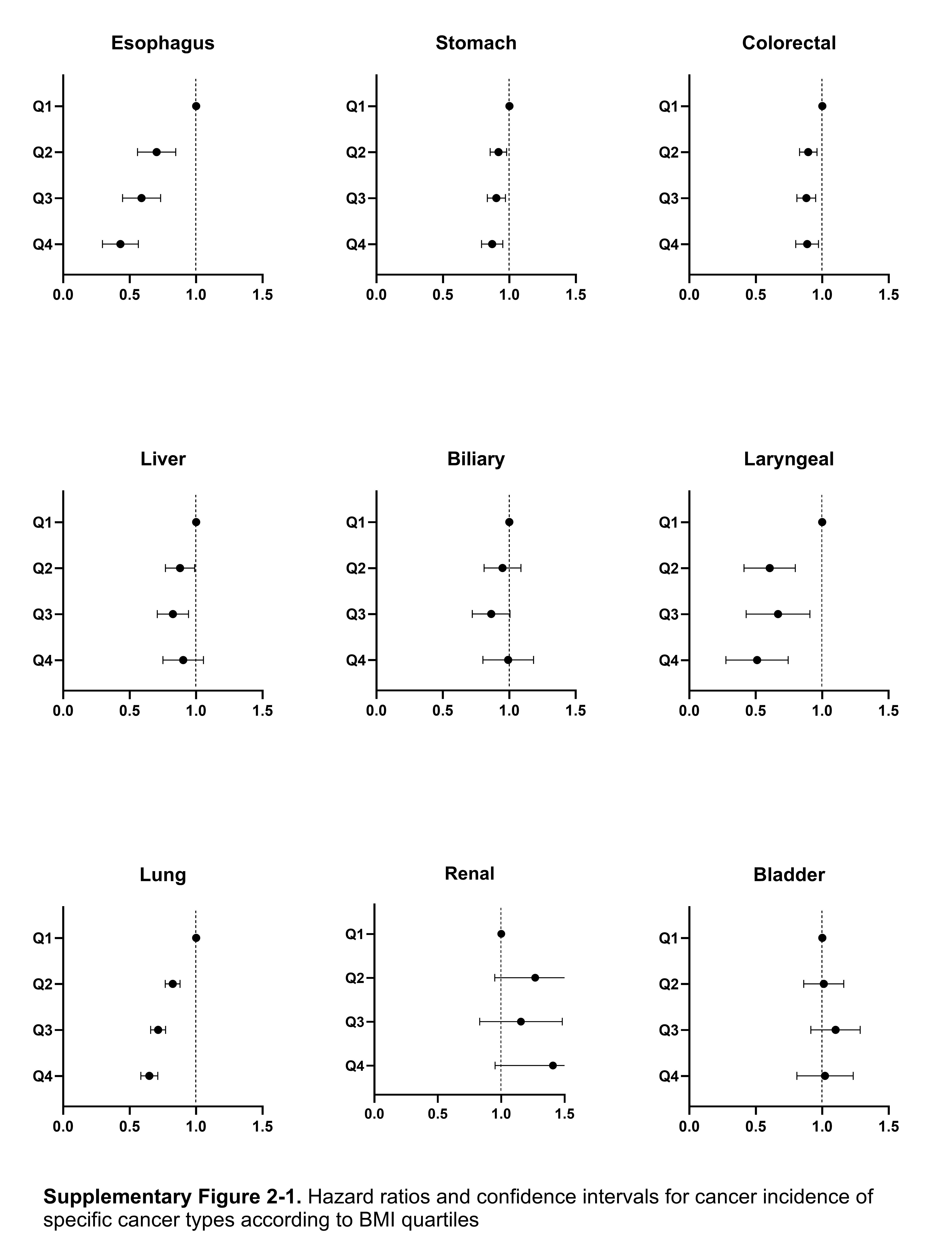

Supplement: Supplementary file 2 [file Image2.jpeg]

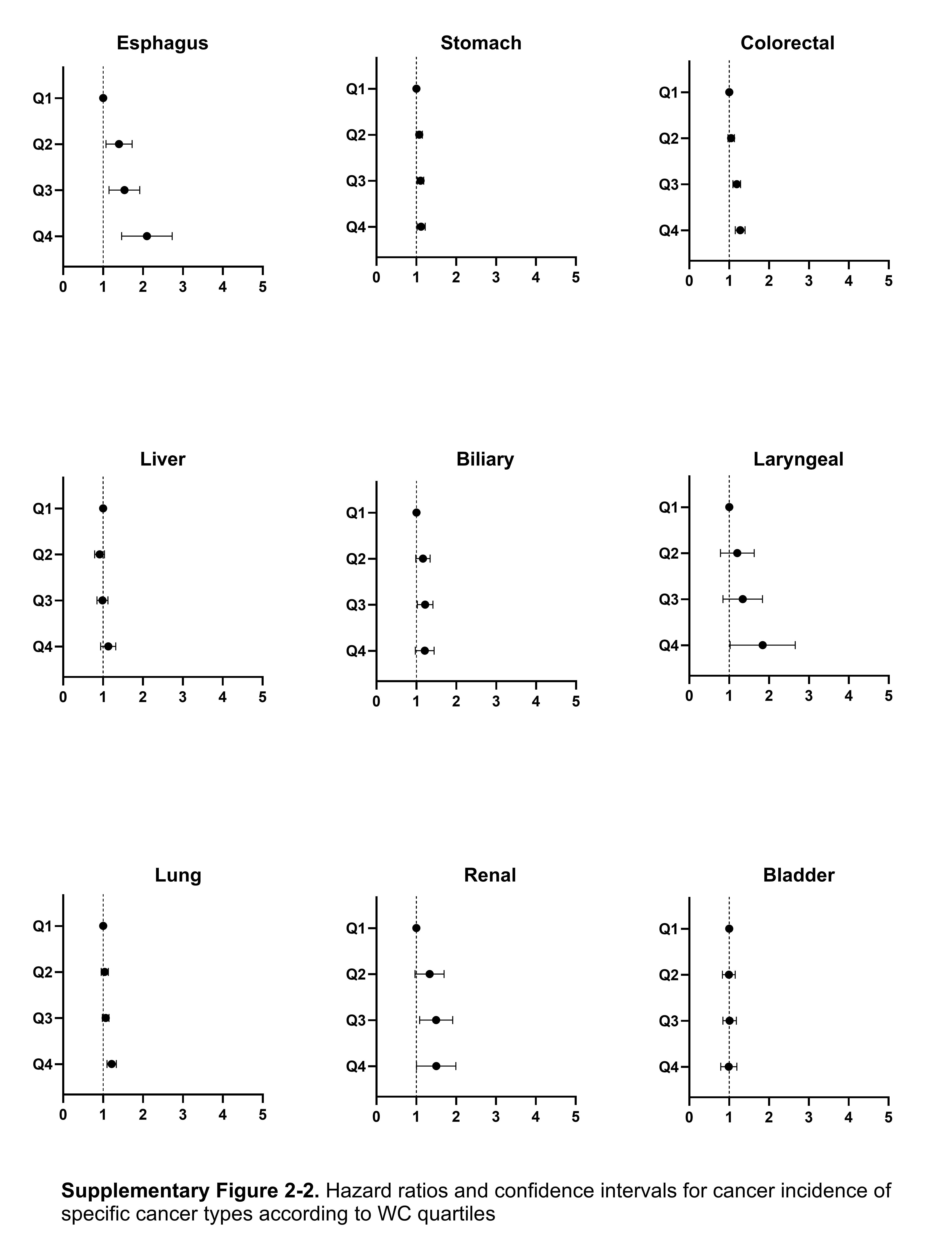

Supplement: Supplementary file 3 [file Image3.jpeg]

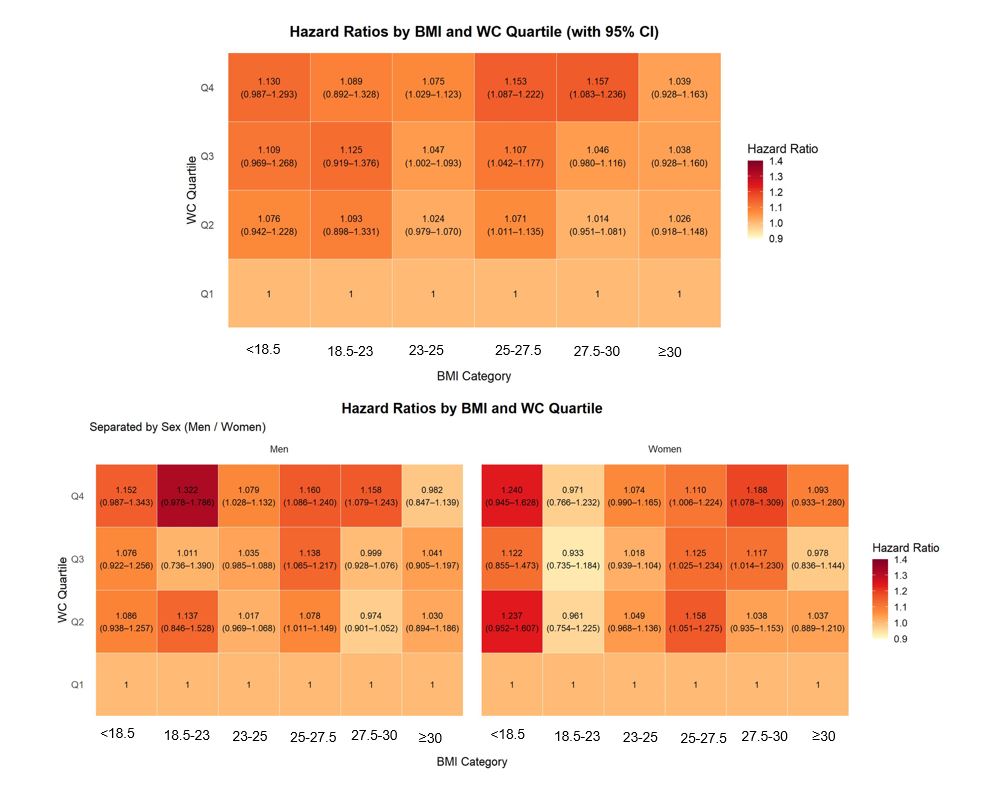

Supplement: Supplementary file 4 [file Image4.jpeg]
