# Supplementary material for: Contrasting associations of body mass index and waist circumference with cancer incidence in the elderly: a nationwide population-based study
Source: Front Oncol. 2025 Sep 25;15:1606686. doi: 10.3389/fonc.2025.1606686 (PMC12507619; doi:10.3389/fonc.2025.1606686)
Supplement: Supplementary file 5 [file Table1.docx]

**
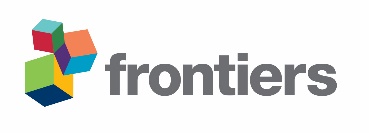
**

| **Supplementary table 1-1.** Baseline characteristics of study participants according to BMI quartiles | | | | | | | | | | | | | | | |
| --- | --- | --- | --- | --- | --- | --- | --- | --- | --- | --- | --- | --- | --- | --- | --- |
|  | Total |  |  |  |  | Men |  |  |  |  | Women |  |  |  |  |
|  | Q1  (n=60,665) | Q2  (n=62,492) | Q3  (n=61,546) | Q4  (n=62,922) | p-value | Q1  (n=31,937) | Q2  (n=31,312) | Q3  (n=31,392) | Q4  (n=31,694) | p-value | Q1  (n=29,835) | Q2  (n=30,230) | Q3  (n=31,173) | Q4  (n=30,052) | p-value |
| Men [n (%)] | 34286(56.5) | 33849 (54.2) | 31670 (51.5) | 26530 (42.2) |  |  |  |  |  |  |  |  |  |  |  |
| Range | 15-21.4 | 21.5-23.4 | 23.5-25.4 | 25.5-39.8 |  | 15-21.2 | 21.3-23.1 | 23.2-25.0 | 25.1-39.8 |  | 15.0 - 21.7 | 21.8 - 23.7 | 23.8 - 25.9 | 26.0 - 39.8 |  |
| Age (years) | 70.6 ± 3.9 | 70 ± 3.7 | 69.8 ± 3.6 | 69.7 ± 3.6 | <0.001 | 70.4 ± 3.8 | 69.9 ± 3.7 | 69.6 ± 3.6 | 69.4 ± 3.5 | <0.001 | 70.9 ± 4 | 70.2 ± 3.8 | 70 ± 3.7 | 70 ± 3.7 | <0.001 |
| BMI (kg/m^2^) | 19.8 ± 1.3 | 22.5 ± 0.6 | 24.4 ± 0.6 | 27.4 ± 1.8 | <0.001 | 19.6 ± 1.3 | 22.3 ± 0.5 | 24.1 ± 0.5 | 26.8 ± 1.6 | <0.001 | 20 ± 1.4 | 22.8 ± 0.6 | 24.8 ± 0.6 | 28.1 ± 1.9 | <0.001 |
| WC (cm) | 74.2 ± 6.1 | 80.1 ± 5.5 | 84.1 ± 5.6 | 89.5 ± 6.7 | <0.001 | 75.3 ± 5.8 | 81.5 ± 4.9 | 85.6 ± 4.9 | 91.2 ± 6 | <0.001 | 72.8 ± 6.1 | 78.5 ± 5.4 | 82.6 ± 5.4 | 88.6 ± 6.6 | <0.001 |
| Systolic BP (mm Hg) | 127.2 ± 17 | 130 ± 16.6 | 131.5 ± 16.3 | 133.6 ± 16.4 | <0.001 | 127.5 ± 17.1 | 130.4 ± 16.7 | 132 ± 16.3 | 134.1 ± 16.2 | <0.001 | 126.8 ± 16.9 | 129.4 ± 16.4 | 131 ± 16.3 | 133.4 ± 16.5 | <0.001 |
| Diastolic BP (mm Hg) | 77.2 ± 10.4 | 78.5 ± 10.2 | 79.3 ± 10.2 | 80.5 ± 10.2 | <0.001 | 77.6 ± 10.5 | 78.9 ± 10.3 | 79.7 ± 10.2 | 81 ± 10.2 | <0.001 | 76.7 ± 10.3 | 78 ± 10.1 | 78.8 ± 10.1 | 80.2 ± 10.2 | <0.001 |
| Fasting glucose (mg/dL) | 96.1 ± 19.7 | 97.4 ± 19.3 | 98.6 ± 19.7 | 100.3 ± 20.6 | <0.001 | 97.2 ± 22 | 98.6 ± 20.8 | 99.9 ± 21.1 | 102 ± 21.7 | <0.001 | 94.6 ± 16.4 | 95.9 ± 17.2 | 97.2 ± 17.9 | 99.2 ± 20 | <0.001 |
| Total cholesterol (mg/dL) | 193.5 ± 42 | 199.6 ± 44.7 | 202.9 ± 46.5 | 205.6 ± 45.1 | <0.001 | 186.3 ± 42.3 | 192.1 ± 40.5 | 195.4 ± 50.5 | 196.3 ± 43.6 | <0.001 | 203 ± 41.5 | 208.5 ± 46.2 | 210.8 ± 43.3 | 212.4 ± 42.6 | <0.001 |
| HDL-C (mg/dL) | 59.3 ± 40.6 | 56.2 ± 39.2 | 54.3 ± 34.5 | 53.6 ± 35 | <0.001 | 59.2 ± 39.9 | 55.8 ± 37.6 | 53.1 ± 33.1 | 51.2 ± 32 | <0.001 | 59.5 ± 41.2 | 56.9 ± 41.2 | 55.7 ± 35.5 | 55.5 ± 37.6 | <0.001 |
| LDL-C (mg/dL) | 115.9 ± 72.9 | 121 ± 61.5 | 124 ± 77.8 | 125.1 ± 73.3 | <0.001 | 109.2 ± 58 | 114.7 ± 57.9 | 117.8 ± 69.2 | 117.2 ± 80 | <0.001 | 124.4 ± 85 | 128.7 ± 65.4 | 130.4 ± 85.5 | 130.9 ± 63 | <0.001 |
| Triglyceride (mg/dL) | 114.5 ± 69.5 | 131.2 ± 79.2 | 143.2 ± 83.5 | 153.2 ± 87.1 | <0.001 | 109.6 ± 71.5 | 127.8 ± 81.3 | 141.4 ± 87 | 156.8 ± 95.1 | <0.001 | 120.3 ± 65.9 | 135 ± 75.9 | 144 ± 79.2 | 150.5 ± 80.3 | <0.001 |
| AST (U/L) | 26.5 ± 17.3 | 25.6 ± 15.1 | 25.6 ± 15.1 | 26.5 ± 19.4 | <0.001 | 27.8 ± 19.7 | 26.6 ± 16.6 | 26.5 ± 13.7 | 27.7 ± 18.6 | <0.001 | 24.9 ± 14.1 | 24.5 ± 15.3 | 24.7 ± 14.1 | 25.7 ± 20.8 | <0.001 |
| ALT (U/L) | 19.4 ± 14.9 | 20.7 ± 15.7 | 22.1 ± 18.5 | 24.4 ± 19.1 | <0.001 | 20.5 ± 15.9 | 21.8 ± 15.8 | 23.5 ± 17 | 26.8 ± 20.3 | <0.001 | 18.1 ± 14.2 | 19.2 ± 15.2 | 20.6 ± 19.1 | 22.7 ± 18.2 | <0.001 |
| Creatinine (mg/dL) | 1 ± 1 | 1 ± 1.2 | 1 ± 1.1 | 1 ± 1.1 | <0.001 | 1.1 ± 1.1 | 1.1 ± 1.2 | 1.1 ± 1.3 | 1.2 ± 1.2 | <0.001 | 0.9 ± 1 | 0.9 ± 0.9 | 0.9 ± 0.9 | 0.9 ± 1.1 | <0.001 |
| eGFR (mL/min/1.73m^2^) | 52.5 ± 16.2 | 58.8 ± 16.6 | 63.2 ± 18.9 | 69.1 ± 20.3 | <0.001 | 55.1 ± 15.4 | 61.3 ± 15.8 | 65.6 ± 19.5 | 71.7 ± 21.1 | <0.001 | 49.2 ± 16.1 | 56 ± 16.3 | 60.8 ± 16.9 | 67.8 ± 20.3 | <0.001 |
| Smoking status  [n (%)] |  |  |  |  | <0.001 |  |  |  |  | <0.001 |  |  |  |  | <0.001 |
| Never | 37048 (61.1) | 41603 (66.6) | 42866 (69.6) | 47614 (75.7) |  | 11346 (35.5) | 12945 (41.3) | 13833 (44.1) | 14771 (46.6) |  | 28125 (94.3) | 29013 (96) | 30078 (96.5) | 29020 (96.6) |  |
| Ex-smoker | 8088 (13.3) | 9334 (14.9) | 9639 (15.7) | 8657 (13.8) |  | 7188 (22.5) | 8252 (26.4) | 9238 (29.4) | 9856 (31.1) |  | 317 (1.1) | 290 (1) | 268 (0.9) | 309 (1) |  |
| Current | 15168 (25) | 11118 (17.8) | 8639 (14) | 6180 (9.8) |  | 13243 (41.5) | 9922 (31.7) | 8134 (25.9) | 6849 (21.6) |  | 1181 (4) | 698 (2.3) | 597 (1.9) | 481 (1.6) |  |
| Unknown | 361 (0.6) | 437 (0.7) | 402 (0.7) | 471 (0.7) |  | 160 (0.5) | 193 (0.6) | 187 (0.6) | 218 (0.7) |  | 212 (0.7) | 229 (0.8) | 230 (0.7) | 242 (0.8) |  |
| Drinking habit  [n (%)] |  |  |  |  | <0.001 |  |  |  |  | <0.001 |  |  |  |  | 0.009 |
| None | 40244 (66.3) | 41642 (66.6) | 41751 (67.8) | 45312 (72) |  | 15491 (48.5) | 14704 (47) | 14827 (47.2) | 15189 (47.9) |  | 26761 (89.7) | 27164 (89.9) | 27891 (89.5) | 26922 (89.6) |  |
| Moderate | 11738 (19.3) | 12372 (19.8) | 12068 (19.6) | 10643 (16.9) |  | 9342 (29.3) | 9799 (31.3) | 10056 (32) | 9949 (31.4) |  | 1879 (6.3) | 1869 (6.2) | 1992 (6.4) | 1935 (6.4) |  |
| Heavy | 7359 (12.1) | 6942 (11.1) | 6211 (10.1) | 5455 (8.7) |  | 6412 (20.1) | 6070 (19.4) | 5747 (18.3) | 5816 (18.4) |  | 529 (1.8) | 442 (1.5) | 476 (1.5) | 475 (1.6) |  |
| Unknown | 1324 (2.2) | 1536 (2.5) | 1516 (2.5) | 1512 (2.4) |  | 692 (2.2) | 739 (2.4) | 762 (2.4) | 740 (2.3) |  | 666 (2.2) | 755 (2.5) | 814 (2.6) | 720 (2.4) |  |
| Exercise  [n (%)] |  |  |  |  | <0.001 |  |  |  |  | <0.001 |  |  |  |  | <0.001 |
| None | 20804 (34.3) | 19268 (30.8) | 18235 (29.6) | 19742 (31.4) |  | 10038 (31.4) | 8663 (27.7) | 8093 (25.8) | 8095 (25.5) |  | 11363 (38.1) | 10417 (34.5) | 10669 (34.2) | 10711 (35.6) |  |
| Irregular | 28746 (47.4) | 29900 (47.8) | 29641 (48.2) | 30100 (47.8) |  | 14815 (46.4) | 14578 (46.6) | 14733 (46.9) | 14839 (46.8) |  | 14500 (48.6) | 14922 (49.4) | 15331 (49.2) | 14669 (48.8) |  |
| Regular | 10746 (17.7) | 12913 (20.7) | 13297 (21.6) | 12678 (20.1) |  | 6889 (21.6) | 7859 (25.1) | 8365 (26.6) | 8560 (27) |  | 3802 (12.7) | 4691 (15.5) | 4989 (16) | 4479 (14.9) |  |
| Unknown | 369 (0.6) | 411 (0.7) | 373 (0.6) | 402 (0.6) |  | 195 (0.6) | 212 (0.7) | 201 (0.6) | 200 (0.6) |  | 170 (0.6) | 200 (0.7) | 184 (0.6) | 193 (0.6) |  |
| CCI [n (%)] |  |  |  |  | <0.001 |  |  |  |  | <0.001 |  |  |  |  | <0.001 |
| 0 | 41997 (69.2) | 43093 (69) | 41684 (67.7) | 41041 (65.2) |  | 22633 (70.9) | 22256 (71.1) | 21882 (69.7) | 21727 (68.6) |  | 20070 (67.3) | 20057 (66.3) | 20395 (65.4) | 18795 (62.5) |  |
| 1 | 14876 (24.5) | 15458 (24.7) | 15757 (25.6) | 17076 (27.1) |  | 7574 (23.7) | 7408 (23.7) | 7692 (24.5) | 7951 (25.1) |  | 7575 (25.4) | 7913 (26.2) | 8409 (27) | 8645 (28.8) |  |
| 2 | 3206 (5.3) | 3320 (5.3) | 3437 (5.6) | 4049 (6.4) |  | 1463 (4.6) | 1425 (4.6) | 1504 (4.8) | 1694 (5.3) |  | 1852 (6.2) | 1870 (6.2) | 2002 (6.4) | 2202 (7.3) |  |
| ≥3 | 586 (1) | 621 (1) | 668 (1.1) | 756 (1.2) |  | 267 (0.8) | 223 (0.7) | 314 (1) | 322 (1) |  | 338 (1.1) | 390 (1.3) | 367 (1.2) | 410 (1.4) |  |
| Income  [n (%)] |  |  |  |  | <0.001 |  |  |  |  | <0.001 |  |  |  |  | 0.001 |
| Medicaid | 355 (0.6) | 292 (0.5) | 295 (0.5) | 356 (0.6) |  | 170 (0.5) | 127 (0.4) | 98 (0.3) | 110 (0.3) |  | 184 (0.6) | 196 (0.6) | 193 (0.6) | 220 (0.7) |  |
| 1-30% | 15172 (25) | 15502 (24.8) | 15029 (24.4) | 15108 (24) |  | 8821 (27.6) | 8722 (27.9) | 8852 (28.2) | 8995 (28.4) |  | 6426 (21.5) | 6387 (21.1) | 6434 (20.6) | 6174 (20.5) |  |
| 31-70% | 18599 (30.7) | 18612 (29.8) | 18058 (29.3) | 18325 (29.1) |  | 10030 (31.4) | 9538 (30.5) | 9250 (29.5) | 9161 (28.9) |  | 8915 (29.9) | 8810 (29.1) | 8966 (28.8) | 8924 (29.7) |  |
| >70% | 25951 (42.8) | 27503 (44) | 27611 (44.9) | 28508 (45.3) |  | 12626 (39.5) | 12658 (40.4) | 12944 (41.2) | 13172 (41.6) |  | 13993 (46.9) | 14525 (48) | 15234 (48.9) | 14421 (48) |  |
| Unknown | 588 (1) | 583 (0.9) | 553 (0.9) | 625 (1) |  | 290 (0.9) | 267 (0.9) | 248 (0.8) | 256 (0.8) |  | 317 (1.1) | 312 (1) | 346 (1.1) | 313 (1) |  |
| Values are presented as number (%), mean ± standard deviation  Abbreviations: BP, blood pressure; HDL-C, high-density lipoprotein cholesterol; LDL-C, low-density lipoprotein cholesterol; AST, aspartate aminotransferase; ALT, alanine aminotransferase; eGFR, estimated glomerular filtration rate; CCI, Charlson comorbidity index | | | | | | | | | | | | | | | |

| **Supplementary table 1-2.** Baseline characteristics of study participants according to WC quartiles | | | | | | | | | | | | | | | |
| --- | --- | --- | --- | --- | --- | --- | --- | --- | --- | --- | --- | --- | --- | --- | --- |
|  | Total |  |  |  |  | Men |  |  |  |  | Women |  |  |  |  |
|  | Q1  (n=62,703) | Q2  (n=55,630) | Q3  (n=68,186) | Q4  (n=61,106) | p-value | Q1  (n=33,186) | Q2  (n=29,988) | Q3  (n=30,766) | Q4  (n=32,395) | p-value | Q1  (n=31,939) | Q2  (n=29,185) | Q3  (n=27,549) | Q4  (n=32,617) | p-value |
| Men [n (%)] | 24483(39.1) | 26636 (47.9) | 37685 (55.3) | 37531 (61.4) |  |  |  |  |  |  |  |  |  |  |  |
| Range | 33-76 | 77-81 | 82-87 | 88-129 |  | 33-78 | 79-83 | 84-88 | 89-129 |  | 36-75 | 76-80 | 81-85 | 86-129 |  |
| Age (years) | 70.3 ± 3.9 | 70.0 ± 3.7 | 69.9 ± 3.7 | 70.0 ± 3.7 | <0.001 | 70.1 ± 3.8 | 69.7 ± 3.6 | 69.6 ± 3.6 | 69.7 ± 3.6 | <0.001 | 70.4 ± 3.9 | 70.2 ± 3.8 | 70.1 ± 3.8 | 70.4 ± 3.8 | <0.001 |
| BMI (kg/m^2^) | 20.8 ± 2.2 | 22.8 ± 2.1 | 24.2 ± 2.1 | 26.4 ± 2.6 | <0.001 | 20.4 ± 2.0 | 22.5 ± 1.8 | 23.9 ± 1.8 | 26.0 ± 2.2 | <0.001 | 21 ± 2.2 | 23.2 ± 2.1 | 24.6 ± 2.1 | 26.9 ± 2.7 | <0.001 |
| WC (cm) | 71.7 ± 3.9 | 79.2 ± 1.4 | 84.4 ± 1.7 | 92.5 ± 4.3 | <0.001 | 73.5 ± 4.0 | 81.1 ± 1.3 | 85.9 ± 1.4 | 93.3 ± 4.1 | <0.001 | 70.6 ± 3.8 | 78.1 ± 1.5 | 82.9 ± 1.4 | 90.8 ± 4.6 | <0.001 |
| Systolic BP (mm Hg) | 127.1 ± 16.9 | 129.9 ± 16.6 | 131.6 ± 16.4 | 133.7 ± 16.5 | <0.001 | 127.6 ± 17.1 | 130.6 ± 16.5 | 132.1 ± 16.5 | 133.9 ± 16.3 | <0.001 | 126.8 ± 16.7 | 129.6 ± 16.5 | 131 ± 16.3 | 133.3 ± 16.6 | <0.001 |
| Diastolic BP (mm Hg) | 77.1 ± 10.3 | 78.5 ± 10.1 | 79.4 ± 10.2 | 80.6 ± 10.3 | <0.001 | 77.6 ± 10.4 | 79.0 ± 10.2 | 79.8 ± 10.2 | 80.8 ± 10.3 | <0.001 | 76.7 ± 10.2 | 78.1 ± 10.1 | 78.9 ± 10.1 | 80.1 ± 10.4 | <0.001 |
| Fasting glucose (mg/dL) | 95.4 ± 17.6 | 97.0 ± 19.0 | 98.7 ± 20.4 | 101.1 ± 21.8 | <0.001 | 96.8 ± 19.9 | 98.6 ± 21.2 | 100.0 ± 21.4 | 102.3 ± 22.9 | <0.001 | 94.6 ± 15.5 | 95.9 ± 17.2 | 97.2 ± 18.8 | 99.2 ± 20 | <0.001 |
| Total cholesterol (mg/dL) | 197.0 ± 40.1 | 200.5 ± 47.8 | 201.7 ± 46.5 | 202.6 ± 44.5 | <0.001 | 186.9 ± 42.5 | 192.3 ± 46.2 | 194.6 ± 43.8 | 196.5 ± 45.1 | <0.001 | 203.6 ± 38 | 208.5 ± 46.2 | 210.7 ± 45.5 | 212.2 ± 44.1 | <0.001 |
| HDL-C (mg/dL) | 59.7 ± 42.2 | 56.7 ± 38.2 | 54.5 ± 36.0 | 52.6 ± 32.6 | <0.001 | 59.1 ± 40.6 | 55.5 ± 36.7 | 53.4 ± 33.7 | 51.3 ± 31.5 | <0.001 | 59.8 ± 42.1 | 56.8 ± 36.9 | 55.8 ± 36.1 | 55 ± 39.6 | <0.001 |
| LDL-C (mg/dL) | 119.3 ± 71.2 | 121.6 ± 62.1 | 122.4 ± 63.5 | 122.8 ± 87.2 | <0.001 | 110.6 ± 59.4 | 114.4 ± 59.1 | 116.5 ± 61.8 | 117.4 ± 83.7 | <0.001 | 125.3 ± 82.1 | 128.2 ± 61.3 | 130.3 ± 68.5 | 131 ± 85.5 | <0.001 |
| Triglyceride (mg/dL) | 114.1 ± 66.3 | 130.3 ± 77.2 | 142.0 ± 83.4 | 155.6 ± 90.8 | <0.001 | 107.1 ± 67.5 | 129.1 ± 82.5 | 142.1 ± 87.3 | 157.9 ± 95.9 | <0.001 | 118.5 ± 64.8 | 135.4 ± 74 | 144.1 ± 79.9 | 152.6 ± 81.8 | <0.001 |
| AST (U/L) | 25.7 ± 15.9 | 25.7 ± 16.3 | 25.9 ± 14.6 | 27.0 ± 20.3 | <0.001 | 27.3 ± 18.7 | 26.9 ± 17.3 | 26.7 ± 14.4 | 27.8 ± 18.3 | <0.001 | 24.9 ± 15.5 | 24.4 ± 12 | 24.8 ± 11.3 | 25.6 ± 22.6 | <0.001 |
| ALT (U/L) | 19.1 ± 14.6 | 20.7 ± 17.5 | 22.1 ± 17.6 | 24.8 ± 18.7 | <0.001 | 20.4 ± 15.6 | 22.2 ± 18.8 | 23.6 ± 17.1 | 26.4 ± 17.9 | <0.001 | 18.3 ± 15.5 | 19.3 ± 13.6 | 20.6 ± 18.4 | 22.3 ± 19.1 | <0.001 |
| Creatinine (mg/dL) | 1.0 ± 0.9 | 1.0 ± 1.2 | 1.0 ± 1.2 | 1.1 ± 1.2 | <0.001 | 1.1 ± 1.0 | 1.1 ± 1.4 | 1.1 ± 1.2 | 1.2 ± 1.2 | <0.001 | 0.9 ± 0.9 | 0.9 ± 1 | 0.9 ± 1 | 0.9 ± 1.1 | <0.001 |
| eGFR (mL/min/1.73m^2^) | 53.3 ± 15.8 | 59.1 ± 18.2 | 62.9 ± 18.2 | 68.5 ± 20.6 | <0.001 | 56.2 ± 15.5 | 61.8 ± 17.7 | 65.3 ± 19.5 | 70.4 ± 20.4 | <0.001 | 51.2 ± 16.1 | 57 ± 17.2 | 60.3 ± 18 | 65.3 ± 20.3 | <0.001 |
| Smoking status  [n (%)] |  |  |  |  | <0.001 |  |  |  |  | <0.001 |  |  |  |  | <0.001 |
| Never | 45698 (72.9) | 38835 (69.8) | 45597 (66.9) | 39001 (63.8) |  | 12796 (38.6) | 12573 (41.9) | 13403 (43.6) | 14123 (43.6) |  | 30382 (95.1) | 27980 (95.9) | 26513 (96.2) | 31361 (96.1) |  |
| Ex-smoker | 5731 (9.1) | 6979 (12.5) | 11029 (16.2) | 11979 (19.6) |  | 7540 (22.7) | 7889 (26.3) | 8998 (29.2) | 10107 (31.2) |  | 276 (0.9) | 289 (1) | 257 (0.9) | 362 (1.1) |  |
| Current | 10884 (17.4) | 9449 (17.0) | 11085 (16.3) | 9687 (15.9) |  | 12682 (38.2) | 9341 (31.1) | 8183 (26.6) | 7942 (24.5) |  | 1059 (3.3) | 689 (2.4) | 561 (2) | 648 (2) |  |
| Unknown | 390 (0.6) | 367 (0.7) | 475 (0.7) | 439 (0.7) |  | 168 (0.5) | 185 (0.6) | 182 (0.6) | 223 (0.7) |  | 222 (0.7) | 227 (0.8) | 218 (0.8) | 246 (0.8) |  |
| Drinking habit  [n (%)] |  |  |  |  | <0.001 |  |  |  |  | <0.001 |  |  |  |  | <0.001 |
| None | 47088 (75.1) | 38487 (69.2) | 44928 (65.9) | 38446 (62.9) |  | 16724 (50.4) | 14009 (46.7) | 14397 (46.8) | 15081 (46.6) |  | 28852 (90.3) | 26172 (89.7) | 24646 (89.5) | 29068 (89.1) |  |
| Moderate | 9305 (14.8) | 10097 (18.2) | 13937 (20.4) | 13482 (22.1) |  | 9653 (29.1) | 9468 (31.6) | 9828 (31.9) | 10197 (31.5) |  | 1891 (5.9) | 1832 (6.3) | 1753 (6.4) | 2199 (6.7) |  |
| Heavy | 4918 (7.8) | 5673 (10.2) | 7658 (11.2) | 7718 (12.6) |  | 6066 (18.3) | 5800 (19.3) | 5803 (18.9) | 6376 (19.7) |  | 512 (1.6) | 430 (1.5) | 428 (1.6) | 552 (1.7) |  |
| Unknown | 1392 (2.2) | 1373 (2.5) | 1663 (2.4) | 1460 (2.4) |  | 743 (2.2) | 711 (2.4) | 738 (2.4) | 741 (2.3) |  | 684 (2.1) | 751 (2.6) | 722 (2.6) | 798 (2.4) |  |
| Exercise  [n (%)] |  |  |  |  | <0.001 |  |  |  |  | <0.001 |  |  |  |  | <0.001 |
| None | 21293 (34.0) | 17416 (31.3) | 20310 (29.8) | 19030 (31.1) |  | 10140 (30.6) | 8211 (27.4) | 7885 (25.6) | 8653 (26.7) |  | 11566 (36.2) | 9982 (34.2) | 9419 (34.2) | 12193 (37.4) |  |
| Irregular | 29935 (47.7) | 26631 (47.9) | 32837 (48.2) | 28984 (47.4) |  | 15198 (45.8) | 13916 (46.4) | 14579 (47.4) | 15272 (47.1) |  | 15592 (48.8) | 14455 (49.5) | 13650 (49.5) | 15725 (48.2) |  |
| Regular | 11128 (17.7) | 11236 (20.2) | 14582 (21.4) | 12688 (20.8) |  | 7659 (23.1) | 7673 (25.6) | 8087 (26.3) | 8254 (25.5) |  | 4609 (14.4) | 4567 (15.6) | 4284 (15.6) | 4501 (13.8) |  |
| Unknown | 347 (0.6) | 347 (0.6) | 457 (0.7) | 404 (0.7) |  | 189 (0.6) | 188 (0.6) | 215 (0.7) | 216 (0.7) |  | 172 (0.5) | 181 (0.6) | 196 (0.7) | 198 (0.6) |  |
| CCI [n (%)] |  |  |  |  | <0.001 |  |  |  |  | <0.001 |  |  |  |  | <0.001 |
| 0 | 43508 (69.4) | 38381 (69.0) | 46133 (67.7) | 39793 (65.1) |  | 23848 (71.9) | 21293 (71) | 21534 (70) | 21823 (67.4) |  | 21757 (68.1) | 19422 (66.5) | 17932 (65.1) | 20206 (61.9) |  |
| 1 | 15270 (24.4) | 13817 (24.8) | 17417 (25.5) | 16663 (27.3) |  | 7661 (23.1) | 7142 (23.8) | 7468 (24.3) | 8354 (25.8) |  | 7961 (24.9) | 7626 (26.1) | 7467 (27.1) | 9488 (29.1) |  |
| 2 | 3333 (5.3) | 2880 (5.2) | 3891 (5.7) | 3908 (6.4) |  | 1414 (4.3) | 1325 (4.4) | 1484 (4.8) | 1863 (5.8) |  | 1889 (5.9) | 1779 (6.1) | 1800 (6.5) | 2458 (7.5) |  |
| ≥3 | 592 (0.9) | 552 (1.0) | 745 (1.1) | 742 (1.2) |  | 263 (0.8) | 228 (0.8) | 280 (0.9) | 355 (1.1) |  | 332 (1) | 358 (1.2) | 350 (1.3) | 465 (1.4) |  |
| Income  [n (%)] |  |  |  |  | <0.001 |  |  |  |  | <0.001 |  |  |  |  | <0.001 |
| Medicaid | 395 (0.6) | 268 (0.5) | 337 (0.5) | 298 (0.5) |  | 188 (0.6) | 114 (0.4) | 97 (0.3) | 106 (0.3) |  | 215 (0.7) | 164 (0.6) | 177 (0.6) | 237 (0.7) |  |
| 1-30% | 14961 (23.9) | 13494 (24.3) | 17079 (25.0) | 15277 (25.0) |  | 9084 (27.4) | 8536 (28.5) | 8775 (28.5) | 8995 (27.8) |  | 6952 (21.8) | 6058 (20.8) | 5723 (20.8) | 6688 (20.5) |  |
| 31-70% | 19096 (30.5) | 16721 (30.1) | 19962 (29.3) | 17815 (29.2) |  | 10450 (31.5) | 9228 (30.8) | 8962 (29.1) | 9339 (28.8) |  | 9480 (29.7) | 8532 (29.2) | 7977 (29) | 9626 (29.5) |  |
| >70% | 27663 (44.1) | 24606 (44.2) | 30166 (44.2) | 27138 (44.4) |  | 13182 (39.7) | 11856 (39.5) | 12679 (41.2) | 13683 (42.2) |  | 14970 (46.9) | 14126 (48.4) | 13388 (48.6) | 15689 (48.1) |  |
| Unknown | 588 (0.9) | 541 (1.0) | 642 (0.9) | 578 (0.9) |  | 282 (0.8) | 254 (0.8) | 253 (0.8) | 272 (0.8) |  | 322 (1) | 305 (1) | 284 (1) | 377 (1.2) |  |
| Values are presented as number (%), mean ± standard deviation  Abbreviations: BP, blood pressure; HDL-C, high-density lipoprotein cholesterol; LDL-C, low-density lipoprotein cholesterol; AST, aspartate aminotransferase; ALT, alanine aminotransferase; eGFR, estimated glomerular filtration rate; CCI, Charlson comorbidity index | | | | | | | | | | | | | | | |

|  | | | | | | | | |  |
| --- | --- | --- | --- | --- | --- | --- | --- | --- | --- |
| **Supplementary table 2-1.** Hazard ratios and confidence intervals for developing cancer according to deciles of BMI | | | | | | | | |  |
| BMI | Range | Events  (n) | Follow-up duration (person-year) | Incidence rate  (per 1000 person-years) | Crude | Model 1 | Model 2 | Model 3 | |
| Total |  |  |  |  |  |  |  |  | |
| Q1  (n=25,446) | 15.0-19.7 | 4863 | 235567.1294 | 20.64379701 | 1.166 (1.119 - 1.215) | 1.104 (1.060 - 1.151) | 1.216 (1.163 - 1.273) | 1.145 (1.094 - 1.198) | |
| Q2  (n=23,234) | 19.8-20.9 | 4273 | 225515.5729 | 18.9476937 | 1.068 (1.024 - 1.115) | 1.032 (0.989 - 1.077) | 1.094 (1.047 - 1.143) | 1.058 (1.012 - 1.106) | |
| Q3  (n=26,982) | 21.0-21.9 | 4817 | 266085.347 | 18.10321408 | 1.020 (0.979 - 1.063) | 1.008 (0.968 - 1.051) | 1.044 (1.001 - 1.089) | 1.021 (0.979 - 1.065) | |
| Q4  (n=23,860) | 22.0-22.7 | 4251 | 237051.9206 | 17.93278025 | 1.010 (0.968 - 1.054) | 1.000 (0.958 - 1.044) | 1.014 (0.972 - 1.059) | 1.006 (0.964 - 1.050) | |
| Q5  (n=23,635) | 22.8-23.4 | 4190 | 236054.5079 | 17.75013762 | 1 | 1 | 1 | 1 | |
| Q6  (n=27,079) | 23.5-24.2 | 4687 | 272780.6845 | 17.18230163 | 0.968 (0.928 - 1.009) | 0.981 (0.941 - 1.023) | 0.965 (0.926 - 1.006) | 0.972 (0.933 - 1.014) | |
| Q7  (n=23,652) | 24.3-25.0 | 4191 | 237937.2731 | 17.61388599 | 0.992 (0.950 - 1.035) | 1.011 (0.969 - 1.055) | 0.979 (0.938 - 1.022) | 0.991 (0.949 - 1.035) | |
| Q8  (n=23,096) | 25.1-25.9 | 3837 | 234066.6064 | 16.39276981 | 0.923 (0.883 - 0.964) | 0.974 (0.933 - 1.018) | 0.928 (0.888 - 0.971) | 0.945 (0.903 - 0.988) | |
| Q9  (n=25,455) | 26.0-27.3 | 4292 | 258810.6037 | 16.58355546 | 0.934 (0.895 - 0.974) | 1.005 (0.964 - 1.049) | 0.939 (0.898 - 0.982) | 0.962 (0.920 - 1.005) | |
| Q10  (n=25,186) | 27.4-39.8 | 3968 | 257214.8939 | 15.4267894 | 0.869 (0.832 - 0.907) | 1.026 (0.982 - 1.072) | 0.917 (0.873 - 0.962) | 0.942 (0.897 - 0.989) | |
| p-value |  |  |  |  | <0.001 | <0.001 | <0.001 | <0.001 | |
| p-for trend |  |  |  |  | <0.001 | <0.001 | <0.001 | <0.001 | |
| Men |  |  |  |  |  |  |  |  | |
| Q1  (n=12,701) | 15.0-19.5 | 3191 | 109060.3778 | 29.25902206 | 1.185 (1.128 - 1.246) | 1.146 (1.090 - 1.205) | 1.294 (1.225 - 1.368) | 1.196 (1.132 - 1.264) | |
| Q2  (n=12,563) | 19.6-20.7 | 3091 | 115139.9726 | 26.84558568 | 1.084 (1.031 - 1.140) | 1.065 (1.012 - 1.120) | 1.147 (1.089 - 1.209) | 1.095 (1.039 - 1.154) | |
| Q3  (n=12,642) | 20.8-21.6 | 3021 | 117524.3094 | 25.70532017 | 1.037 (0.986 - 1.091) | 1.027 (0.976 - 1.08) | 1.074 (1.020 - 1.130) | 1.041 (0.988 - 1.095) | |
| Q4  (n=12,612) | 21.7-22.4 | 2965 | 118823.05 | 24.95307098 | 1.006 (0.956 - 1.059) | 1.002 (0.952 - 1.054) | 1.022 (0.972 - 1.076) | 1.008 (0.957 - 1.060) | |
| Q5  (n=12,731) | 22.5-23.1 | 2994 | 120684.5996 | 24.80846778 | 1 | 1 | 1 | 1 | |
| Q6  (n=11,939) | 23.2-23.8 | 2753 | 114310.0151 | 24.08362906 | 0.971 (0.922 - 1.022) | 0.977 (0.928 - 1.029) | 0.959 (0.911 - 1.011) | 0.967 (0.918 - 1.018) | |
| Q7  (n=13,213) | 23.9-24.6 | 3090 | 127158.9706 | 24.30029109 | 0.979 (0.931 - 1.029) | 0.988 (0.940 - 1.039) | 0.951 (0.904 - 1.000) | 0.968 (0.920 - 1.018) | |
| Q8  (n=12,774) | 24.7-25.5 | 2913 | 123226.7187 | 23.63935379 | 0.952 (0.905 - 1.002) | 0.965 (0.917 - 1.016) | 0.910 (0.863 - 0.958) | 0.933 (0.886 - 0.983) | |
| Q9  (n=12,877) | 25.6-26.8 | 2918 | 124793.6016 | 23.38260906 | 0.941 (0.895 - 0.991) | 0.957 (0.909 - 1.007) | 0.879 (0.834 - 0.928) | 0.912 (0.865 - 0.962) | |
| Q10  (n=12,283) | 26.9-39.8 | 2860 | 118443.0664 | 24.1466224 | 0.973 (0.924 - 1.024) | 0.992 (0.943 - 1.045) | 0.863 (0.815 - 0.915) | 0.907 (0.856 - 0.961) | |
| p-value |  |  |  |  | <0.001 | <0.001 | <0.001 | <0.001 | |
| p-for trend |  |  |  |  | <0.001 | <0.001 | <0.001 | <0.001 | |
| Women |  |  |  |  |  |  |  |  | |
| Q1  (n=12,046) | 15.0-19.9 | 1321 | 121741.1992 | 10.85088704 | 1.018 (0.942 - 1.099) | 0.978 (0.905 - 1.057) | 1.042 (0.959 - 1.131) | 1.026 (0.945 - 1.115) | |
| Q2  (n=12,383) | 20.0-21.2 | 1323 | 128553.5387 | 10.29143199 | 0.964 (0.893 - 1.041) | 0.944 (0.874 - 1.020) | 0.978 (0.904 - 1.059) | 0.972 (0.898 - 1.052) | |
| Q3  (n=12,880) | 21.3-22.2 | 1399 | 135591.6222 | 10.31774661 | 0.966 (0.896 - 1.043) | 0.957 (0.887 - 1.032) | 0.977 (0.905 - 1.055) | 0.975 (0.903 - 1.053) | |
| Q4  (n=11,497) | 22.3-23.0 | 1266 | 120892.8487 | 10.47208345 | 0.981 (0.907 - 1.060) | 0.974 (0.901 - 1.053) | 0.984 (0.910 - 1.063) | 0.983 (0.909 - 1.062) | |
| Q5  (n=11,259) | 23.1-23.7 | 1270 | 118928.3669 | 10.67869705 | 1 | 1 | 1 | 1 | |
| Q6  (n=13,173) | 23.8-24.6 | 1441 | 139153.6975 | 10.35545606 | 0.970 (0.899 - 1.045) | 0.972 (0.901 - 1.048) | 0.963 (0.893 - 1.038) | 0.963 (0.893 - 1.039) | |
| Q7  (n=11,660) | 24.7-25.4 | 1363 | 122922.6229 | 11.08827625 | 1.038 (0.962 - 1.121) | 1.040 (0.964 - 1.123) | 1.019 (0.944 - 1.101) | 1.021 (0.945 - 1.103) | |
| Q8  (n=11,887) | 25.5-26.4 | 1332 | 125640.5229 | 10.60167507 | 0.993 (0.919 - 1.072) | 0.996 (0.922 - 1.075) | 0.965 (0.892 - 1.043) | 0.967 (0.894 - 1.046) | |
| Q9  (n=12,299) | 26.5-27.9 | 1404 | 129934.7461 | 10.80542382 | 1.012 (0.938 - 1.092) | 1.016 (0.942 - 1.096) | 0.971 (0.897 - 1.051) | 0.973 (0.899 - 1.053) | |
| Q10  (n=12,206) | 28.0-39.8 | 1454 | 128560.6927 | 11.30983328 | 1.059 (0.982 - 1.142) | 1.064 (0.986 - 1.147) | 0.986 (0.907 - 1.072) | 0.989 (0.910 - 1.076) | |
| p-value |  |  |  |  | 0.178 | 0.044 | 0.645 | 0.758 | |
| p-for trend |  |  |  |  | 0.043 | 0.001 | 0.541 | 10 | |
| Abbreviation: BMI, body mass index  Model 1: adjusted for age, sex  Model 2: adjusted for model 1 plus waist circumference  Model 3: adjusted for model 2 plus alcohol, smoking, exercise, fasting blood glucose, income, charlson comorbidity index  In sex-stratified analyses, sex was excluded from the covariates. | | | | | | | | |  |

|  | | | | | | | | |  |
| --- | --- | --- | --- | --- | --- | --- | --- | --- | --- |
| **Supplementary table 2-2.** Hazard ratios and confidence intervals for developing cancer according to deciles of WC | | | | | | | | |  |
| WC | Range | Events  (n) | Follow-up duration (person-year) | Incidence rate  (per 1000 person-years) | Crude | Model 1 | Model 2 | Model 3 | |
| Total |  |  |  |  |  |  |  |  | |
| Q1  (n=25,140) | 33-71 | 3706 | 248478.705 | 14.91475899 | 0.838 (0.795 - 0.882) | 0.954 (0.905 - 1.005) | 0.871 (0.824 - 0.920) | 0.880 (0.833 - 0.930) | |
| Q2  (n=26,937) | 72-75 | 4405 | 267211.384 | 16.4850761 | 0.926 (0.880 - 0.973) | 1.009 (0.959 - 1.061) | 0.958 (0.910 - 1.008) | 0.960 (0.912 - 1.011) | |
| Q3  (n=19,197) | 76-77 | 3093 | 192244.5175 | 16.08888535 | 0.903 (0.856 - 0.953) | 0.979 (0.927 - 1.033) | 0.946 (0.896 - 0.999) | 0.948 (0.897 - 1.001) | |
| Q4  (n=34,152) | 78-80 | 5750 | 341935.1814 | 16.81605261 | 0.944 (0.899 - 0.991) | 0.988 (0.941 - 1.037) | 0.975 (0.929 - 1.024) | 0.973 (0.927 - 1.021) | |
| Q5  (n=12,907) | 81-81 | 2296 | 128886.6201 | 17.81410668 | 1 | 1 | 1 | 1 | |
| Q6  (n=35,612) | 82-84 | 6294 | 355373.8426 | 17.71092648 | 0.994 (0.948 - 1.043) | 0.989 (0.943 - 1.038) | 1.005 (0.958 - 1.054) | 1.002 (0.955 - 1.051) | |
| Q7  (n=23,007) | 85-86 | 4253 | 228295.091 | 18.62939751 | 1.046 (0.994 - 1.100) | 1.004 (0.954 - 1.056) | 1.039 (0.987 - 1.094) | 1.032 (0.981 - 1.086) | |
| Q8  (n=18,365) | 87-88 | 3399 | 183087.7947 | 18.56486396 | 1.042 (0.988 - 1.099) | 0.995 (0.944 - 1.049) | 1.044 (0.989 - 1.101) | 1.036 (0.982 - 1.093) | |
| Q9  (n=28,260) | 89-92 | 5383 | 279710.883 | 19.2448715 | 1.081 (1.029 - 1.135) | 1.003 (0.955 - 1.053) | 1.074 (1.021 - 1.130) | 1.055 (1.003 - 1.110) | |
| Q10  (n=24,048) | 93-129 | 4790 | 235860.5202 | 20.30861289 | 1.141 (1.086 - 1.199) | 1.048 (0.998 - 1.102) | 1.176 (1.114 - 1.242) | 1.137 (1.076 - 1.200) | |
| p-value |  |  |  |  | <0.001 | 0.009 | <0.001 | <0.001 | |
| p-for trend |  |  |  |  | <0.001 | 0.001 | <0.001 | <0.001 | |
| Men |  |  |  |  |  |  |  |  | |
| Q1  (n=13,800) | 33-73 | 3244 | 124638.5489 | 26.02726065 | 1.059 (1.007 - 1.113) | 1.028 (0.977 - 1.081) | 0.892 (0.844 - 0.942) | 0.905 (0.857 - 0.957) | |
| Q2  (n=10,683) | 74-76 | 2521 | 98543.34839 | 25.58265008 | 1.039 (0.984 - 1.096) | 1.025 (0.971 - 1.082) | 0.938 (0.888 - 0.992) | 0.948 (0.896 - 1.002) | |
| Q3  (n=13,158) | 77-79 | 3063 | 123361.4894 | 24.82946676 | 1.007 (0.957 - 1.060) | 1.001 (0.951 - 1.054) | 0.947 (0.900 - 0.998) | 0.951 (0.903 - 1.001) | |
| Q4  (n=13,478) | 80-81 | 3145 | 127564.7995 | 24.65413667 | 1.000 (0.950 - 1.052) | 0.997 (0.948 - 1.049) | 0.973 (0.925 - 1.024) | 0.979 (0.930 - 1.030) | |
| Q5  (n=12,055) | 82-83 | 2820 | 114342.82 | 24.66267668 | 1 | 1 | 1 | 1 | |
| Q6  (n=12,746) | 84-85 | 2966 | 121211.2635 | 24.46967315 | 0.992 (0.943 - 1.045) | 0.994 (0.944 - 1.046) | 1.017 (0.966 - 1.071) | 1.018 (0.967 - 1.072) | |
| Q7  (n=12,884) | 86-87 | 3030 | 123011.7919 | 24.63178491 | 0.998 (0.948 - 1.051) | 1.003 (0.953 - 1.056) | 1.052 (0.999 - 1.108) | 1.046 (0.993 - 1.101) | |
| Q8  (n=9,809) | 88-89 | 2290 | 93979.68789 | 24.3669675 | 0.988 (0.935 - 1.044) | 0.990 (0.937 - 1.046) | 1.061 (1.003 - 1.123) | 1.052 (0.994 - 1.113) | |
| Q9  (n=15,407) | 90-83 | 3677 | 146597.9384 | 25.08220811 | 1.017 (0.968 - 1.068) | 1.019 (0.971 - 1.071) | 1.130 (1.074 - 1.190) | 1.104 (1.048 - 1.162) | |
| Q10  (n=12,315) | 94-129 | 3040 | 115912.9938 | 26.22656787 | 1.065 (1.012 - 1.121) | 1.058 (1.006 - 1.114) | 1.261 (1.190 - 1.336) | 1.201 (1.133 - 1.273) | |
| p-value |  |  |  |  | 0.021 | 0.26 | <0.001 | <0.001 | |
| p-for trend |  |  |  |  | 0.6 | 0.502 | <0.001 | <0.001 | |
| Women |  |  |  |  |  |  |  |  | |
| Q1  (n=13,207) | 36-70 | 1365 | 136606.7406 | 9.992186287 | 0.943 (0.876 - 1.017) | 0.932 (0.864 - 1.004) | 0.922 (0.851 - 0.999) | 0.923 (0.852 - 1.000) | |
| Q2  (n=9,917) | 71-73 | 1050 | 103892.1561 | 10.10663403 | 0.954 (0.880 - 1.033) | 0.953 (0.880 - 1.033) | 0.947 (0.873 - 1.028) | 0.950 (0.875 - 1.031) | |
| Q3  (n=15,096) | 74-76 | 1638 | 158316.345 | 10.34637327 | 0.976 (0.909 - 1.049) | 0.975 (0.908 - 1.048) | 0.972 (0.904 - 1.045) | 0.974 (0.906 - 1.047) | |
| Q4  (n=10,431) | 77-78 | 1116 | 110299.4771 | 10.11790835 | 0.955 (0.882 - 1.033) | 0.952 (0.880 - 1.030) | 0.950 (0.878 - 1.028) | 0.950 (0.878 - 1.028) | |
| Q5  (n=12,473) | 79-80 | 1392 | 131340.9583 | 10.59836945 | 1 | 1 | 1 | 1 | |
| Q6  (n=12,043) | 81-82 | 1400 | 126619.1567 | 11.05677874 | 1.043 (0.969 - 1.124) | 1.045 (0.970 - 1.126) | 1.047 (0.972 - 1.128) | 1.048 (0.973 - 1.129) | |
| Q7  (n=10,788) | 83-84 | 1216 | 113475.4278 | 10.71597635 | 1.011 (0.936 - 1.092) | 1.011 (0.936 - 1.092) | 1.014 (0.939 - 1.096) | 1.013 (0.937 - 1.094) | |
| Q8  (n=13,760) | 85-87 | 1548 | 144890.4559 | 10.68393353 | 1.008 (0.938 - 1.084) | 1.005 (0.935 - 1.081) | 1.011 (0.939 - 1.088) | 1.008 (0.936 - 1.086) | |
| Q9  (n=12,278) | 88-91 | 1468 | 128493.3005 | 11.42472016 | 1.078 (1.002 - 1.160) | 1.072 (0.996 - 1.153) | 1.080 (1.000 - 1.166) | 1.074 (0.995 - 1.159) | |
| Q10  (n=11,297) | 92-129 | 1380 | 117985.8398 | 11.69631883 | 1.104 (1.025 - 1.190) | 1.087 (1.009 - 1.171) | 1.102 (1.014 - 1.197) | 1.089 (1.002 - 1.184) | |
| p-value |  |  |  |  | <0.001 | <0.001 | 0.011 | 0.023 | |
| p-for trend |  |  |  |  | <0.001 | <0.001 | <0.001 | <0.001 | |
| Abbreviation: WC, waist circumference  Model 1: adjusted for age, sex  Model 2: adjusted for model 1 plus body mass index  Model 3: adjusted for model 2 plus alcohol, smoking, exercise, fasting blood glucose, income, charlson comorbidity index  In sex-stratified analyses, sex was excluded from the covariates | | | | | | | | |  |

| **Supplementary table 3-1.** Hazard ratios and 95% confidence intervals for developing specific cancer types according to BMI quartiles | | | | | | | | | | | | |
| --- | --- | --- | --- | --- | --- | --- | --- | --- | --- | --- | --- | --- |
| BMI,kg/m^2^ | Oral | Esophagus | Stomach | Colorectal | Liver | Biliary | Pancreas | Laryngeal | Lung | Breast | Cervical | Corpus |
| Total (n) | 505 | 711 | 7728 | 6772 | 2207 | 1656 | 1487 | 310 | 6968 |  |  |  |
| Q1 | 1 | 1 | 1 | 1 | 1 | 1 | 1 | 1 | 1 |  |  |  |
| Q2 | 0.613 (0.473 - 0.794) | 0.693 (0.564 - 0.851) | 0.916 (0.857 - 0.980) | 0.893 (0.830 - 0.961) | 0.875 (0.772 - 0.992) | 0.943 (0.814 - 1.091) | 0.911 (0.781 - 1.062) | 0.585 (0.423 - 0.808) | 0.823 (0.769 - 0.880) |  |  |  |
| Q3 | 0.719 (0.541 - 0.957) | 0.578 (0.453 - 0.739) | 0.901 (0.835 - 0.971) | 0.878 (0.810 - 0.952) | 0.820 (0.711 - 0.946) | 0.856 (0.726 - 1.010) | 0.947 (0.798 - 1.123) | 0.639 (0.444 - 0.921) | 0.713 (0.659 - 0.772) |  |  |  |
| Q4 | 0.629 (0.440 - 0.899) | 0.416 (0.303 - 0.572) | 0.868 (0.792 - 0.952) | 0.884 (0.802 - 0.973) | 0.895 (0.755 - 1.060) | 0.980 (0.808 - 1.189) | 0.914 (0.744 - 1.123) | 0.474 (0.296 - 0.761) | 0.646 (0.586 - 0.713) |  |  |  |
| p-value | 0.003 | <0.001 | 0.015 | 0.007 | 0.032 | 0.143 | 0.681 | 0.004 | <0.001 |  |  |  |
| p-for trend | 0.03 | <0.001 | 0.004 | 0.019 | 0.154 | 0.625 | 0.527 | 0.005 | <0.001 |  |  |  |
| Men (n) | 383 | 670 | 5611 | 4362 | 1602 | 993 | 855 | 296 | 5369 |  |  |  |
| Q1 | 1 | 1 | 1 | 1 | 1 | 1 | 1 | 1 | 1 |  |  |  |
| Q2 | 0.598 (0.444 - 0.806) | 0.678 (0.546 - 0.843) | 0.934 (0.863 - 1.011) | 0.894 (0.814 - 0.981) | 0.769 (0.661 - 0.895) | 0.889 (0.738 - 1.072) | 0.82 (0.668 - 1.007) | 0.505 (0.358 - 0.713) | 0.807 (0.747 - 0.871) |  |  |  |
| Q3 | 0.695 (0.501 - 0.964) | 0.585 (0.454 - 0.752) | 0.922 (0.843 - 1.008) | 0.893 (0.806 - 0.989) | 0.778 (0.658 - 0.919) | 0.77 (0.621 - 0.955) | 0.897 (0.716 - 1.123) | 0.63 (0.436 - 0.91) | 0.667 (0.61 - 0.731) |  |  |  |
| Q4 | 0.543 (0.359 - 0.822) | 0.455 (0.332 - 0.623) | 0.889 (0.798 - 0.991) | 0.879 (0.778 - 0.993) | 0.775 (0.636 - 0.946) | 0.84 (0.652 - 1.083) | 0.758 (0.575 - 1) | 0.455 (0.285 - 0.726) | 0.588 (0.526 - 0.657) |  |  |  |
| p-value | 0.001 | <0.001 | 0.178 | 0.084 | 0.004 | 0.121 | 0.137 | 0.001 | <0.001 |  |  |  |
| p-for trend | 0.012 | <0.001 | 0.043 | 0.069 | 0.029 | 0.109 | 0.124 | 0.004 | <0.001 |  |  |  |
| Women (n) | 122 | 41 | 2117 | 2410 | 605 | 663 | 632 | 14 | 1599 | 938 | 360 | 111 |
| Q1 | 1 | 1 | 1 | 1 | 1 | 1 | 1 | 1 | 1 | 1 | 1 | 1 |
| Q2 | 0.748 (0.42 - 1.33) | 0.872 (0.387 - 1.961) | 0.887 (0.78 - 1.008) | 0.921 (0.815 - 1.04) | 1.038 (0.819 - 1.316) | 1.032 (0.815 - 1.306) | 1.04 (0.822 - 1.315) | 2.971 (0.481 - 18.334) | 0.959 (0.83 - 1.107) | 1.598 (1.267 - 2.015) | 1.002 (0.738 - 1.36) | 1.181 (0.621 - 2.247) |
| Q3 | 1.085 (0.609 - 1.933) | 0.414 (0.15 - 1.141) | 0.921 (0.802 - 1.058) | 0.925 (0.811 - 1.056) | 0.987 (0.757 - 1.287) | 0.989 (0.766 - 1.277) | 1.032 (0.797 - 1.337) | 4.088 (0.601 - 27.821) | 0.868 (0.738 - 1.019) | 1.956 (1.539 - 2.487) | 0.791 (0.559 - 1.118) | 1.604 (0.828 - 3.11) |
| Q4 | 1.095 (0.55 - 2.179) | 0.248 (0.069 - 0.892) | 0.813 (0.688 - 0.961) | 0.969 (0.829 - 1.132) | 1.09 (0.797 - 1.49) | 1.054 (0.783 - 1.42) | 1.085 (0.798 - 1.476) | 3.033 (0.29 - 31.74) | 0.83 (0.683 - 1.007) | 2.585 (1.973 - 3.389) | 0.835 (0.557 - 1.253) | 2.604  (1.24 - 5.468) |
| p-value | 0.528 | 0.092 | 0.075 | 0.453 | 0.876 | 0.947 | 0.96 | 0.532 | 0.194 | <0.001 | 0.426 | 0.039 |
| p-for trend | 0.528 | 0.016 | 0.041 | 0.749 | 0.712 | 0.825 | 0.647 | 0.346 | 0.035 | <0.001 | 0.219 | 0.006 |
| BMI,kg/m^2^ | Ovary | Prostate | Testicular | Renal | Bladder | CNS | Thyroid | Hodgikin | non-Hodgikin | Multiple myeloma | Leukemia | Etc. |
| Total (n) |  |  |  | 620 | 1555 | 417 | 999 | 14 | 792 | 375 | 473 | 3723 |
| Q1 |  |  |  | 1 | 1 | 1 | 1 | 1 | 1 | 1 | 1 | 1 |
| Q2 |  |  |  | 1.242 (0.964 - 1.600) | 1.004 (0.864 - 1.166) | 0.939 (0.701 - 1.257) | 1.103 (0.898 - 1.355) | 1.209 (0.218 - 6.699) | 1.037 (0.837 - 1.285) | 0.972 (0.710 - 1.329) | 0.817 (0.624 - 1.070) | 0.921 (0.837 - 1.014) |
| Q3 |  |  |  | 1.125 (0.847 - 1.493) | 1.090 (0.920 - 1.290) | 0.870 (0.630 - 1.202) | 1.262 (1.016 - 1.568) | 0.948 (0.121 - 7.405) | 0.923 (0.725 - 1.176) | 1.051 (0.745 - 1.482) | 0.810 (0.599 - 1.095) | 0.898 (0.806 - 1.000) |
| Q4 |  |  |  | 1.359 (0.979 - 1.887) | 1.006 (0.817 - 1.240) | 0.705 (0.476 - 1.043) | 1.269 (0.986 - 1.632) | 3.459 (0.441 - 27.164) | 1.003 (0.755 - 1.334) | 1.104 (0.734 - 1.660) | 0.763 (0.530 - 1.099) | 0.813 (0.713 - 0.926) |
| p-value |  |  |  | 0.184 | 0.583 | 0.297 | 0.163 | 0.417 | 0.705 | 0.905 | 0.421 | 0.021 |
| p-for trend |  |  |  | 0.154 | 0.703 | 0.078 | 0.041 | 0.247 | 0.78 | 0.559 | 0.178 | 0.003 |
| Men (n) |  | 4381 | 12 | 438 | 1311 | 221 | 203 | 10 | 512 | 206 | 283 | 2053 |
| Q1 |  | 1 | 1 | 1 | 1 | 1 | 1 | 1 | 1 | 1 | 1 | 1 |
| Q2 |  | 1.205 (1.097 - 1.325) | 1.129 (0.22 - 5.794) | 1.113 (0.82 - 1.51) | 0.967 (0.82 - 1.14) | 0.769 (0.514 - 1.153) | 1.269 (0.802 - 2.007) | 1.149 (0.199 - 6.647) | 1.172 (0.891 - 1.543) | 0.634 (0.406 - 0.989) | 0.907 (0.637 - 1.29) | 0.907 (0.796 - 1.033) |
| Q3 |  | 1.245 (1.122 - 1.382) | 0.497 (0.061 - 4.047) | 1.032 (0.736 - 1.446) | 0.947 (0.786 - 1.14) | 0.79 (0.508 - 1.228) | 1.539 (0.939 - 2.522) | 0 (0 - .) | 1.056 (0.775 - 1.439) | 0.788 (0.496 - 1.251) | 0.864 (0.582 - 1.282) | 0.866 (0.747 - 1.003) |
| Q4 |  | 1.154 (1.018 - 1.308) | 0.673 (0.065 - 6.931) | 1.265 (0.858 - 1.866) | 0.971 (0.777 - 1.214) | 0.584 (0.338 - 1.01) | 1.809 (1.015 - 3.225) | 1.792 (0.168 - 19.081) | 1.248 (0.871 - 1.79) | 0.842 (0.487 - 1.454) | 0.666 (0.409 - 1.085) | 0.816 (0.683 - 0.976) |
| p-value |  | <0.001 | 0.832 | 0.482 | 0.942 | 0.262 | 0.224 | 0.966 | 0.458 | 0.213 | 0.379 | 0.154 |
| p-for trend |  | 0.047 | 0.575 | 0.318 | 0.767 | 0.086 | 0.037 | 0.853 | 0.377 | 0.827 | 0.118 | 0.027 |
| Women (n) | 221 |  |  | 182 | 244 | 196 | 796 | 4 | 280 | 169 | 190 | 1670 |
| Q1 | 1 |  |  | 1 | 1 | 1 | 1 | 1 | 1 | 1 | 1 | 1 |
| Q2 | 1.053 (0.716 - 1.547) |  |  | 1.821 (1.122 - 2.955) | 1.425 (0.952 - 2.134) | 1.213 (0.789 - 1.865) | 1.119 (0.894 - 1.401) | 1.409  (0 - .) | 0.837 (0.585 - 1.196) | 1.679 (1.076 - 2.619) | 0.784 (0.513 - 1.199) | 1.003 (0.87 - 1.155) |
| Q3 | 0.853 (0.553 - 1.316) |  |  | 1.425 (0.834 - 2.433) | 1.752 (1.143 - 2.684) | 1.025 (0.637 - 1.65) | 1.315 (1.038 - 1.665) | >999  (0 - .) | 0.818 (0.556 - 1.202) | 1.257 (0.744 - 2.125) | 0.811 (0.51 - 1.29) | 0.895 (0.764 - 1.048) |
| Q4 | 0.65 (0.381 - 1.109) |  |  | 1.646 (0.897 - 3.022) | 1.487 (0.887 - 2.493) | 0.948 (0.539 - 1.666) | 1.218 (0.918 - 1.616) | >999  (0 - .) | 0.834 (0.53 - 1.315) | 1.514 (0.82 - 2.796) | 0.905 (0.523 - 1.567) | 0.857 (0.709 - 1.035) |
| p-value | 0.207 |  |  | 0.102 | 0.074 | 0.653 | 0.134 | 0.715 | 0.734 | 0.122 | 0.645 | 0.201 |
| p-for trend | 0.081 |  |  | 0.333 | 0.098 | 0.652 | 0.1 | 0.06 | 0.461 | 0.412 | 0.751 | 0.056 |
| Abbreviations: BMI, body mass index  Adjusted for age, sex, waist circumference, alcohol, smoking, exercise, fasting glucose, income, charlson comorbidity index  Sex-specific cancers are labeled to each sex | | | | | | | | | | | | |

| **Supplementary table 3-2.** Hazard ratios and 95% confidence intervals for developing specific cancer types according to WC quartiles | | | | | | | | | | | | |
| --- | --- | --- | --- | --- | --- | --- | --- | --- | --- | --- | --- | --- |
| WC, cm | Oral | Esophagus | Stomach | Colorectal | Liver | Biliary | Pancreas | Laryngeal | Lung | Breast | Cervical | Corpus |
| Total (n) | 505 | 711 | 7728 | 6772 | 2207 | 1656 | 1487 | 310 | 6968 |  |  |  |
| Q1 | 1 | 1 | 1 | 1 | 1 | 1 | 1 | 1 | 1 |  |  |  |
| Q2 | 0.790 (0.602 - 1.038) | 1.374 (1.087 - 1.738) | 1.073 (1.000 - 1.151) | 1.047 (0.969 - 1.132) | 0.908 (0.793 - 1.039) | 1.155 (0.990 - 1.346) | 1.045 (0.894 - 1.221) | 1.154 (0.808 - 1.648) | 1.009 (0.939 - 1.085) |  |  |  |
| Q3 | 0.914 (0.689 - 1.213) | 1.502 (1.167 - 1.933) | 1.103 (1.024 - 1.188) | 1.186 (1.095 - 1.285) | 0.978 (0.851 - 1.125) | 1.207 (1.028 - 1.418) | 0.959 (0.811 - 1.135) | 1.278 (0.876 - 1.864) | 1.064 (0.984 - 1.149) |  |  |  |
| Q4 | 0.999 (0.704 - 1.417) | 2.035 (1.499 - 2.761) | 1.117 (1.020 - 1.224) | 1.271 (1.154 - 1.400) | 1.120 (0.947 - 1.325) | 1.194 (0.981 - 1.453) | 1.038 (0.846 - 1.273) | 1.718 (1.089 - 2.711) | 1.211 (1.101 - 1.332) |  |  |  |
| p-value | 0.258 | <0.001 | 0.061 | <0.001 | 0.022 | 0.134 | 0.633 | 0.111 | <0.001 |  |  |  |
| p-for trend | 0.856 | <0.001 | 0.016 | <0.001 | 0.124 | 0.073 | 0.994 | 0.023 | <0.001 |  |  |  |
| Men (n) | 383 | 670 | 5611 | 4362 | 1602 | 993 | 855 | 296 | 5369 |  |  |  |
| Q1 | 1 | 1 | 1 | 1 | 1 | 1 | 1 | 1 | 1 |  |  |  |
| Q2 | 1.177 (0.875 - 1.583) | 1.188 (0.948 - 1.488) | 1.029 (0.951 - 1.113) | 1.128 (1.027 - 1.238) | 1.026 (0.88 - 1.196) | 1.264 (1.046 - 1.528) | 1.112 (0.91 - 1.359) | 1.155 (0.82 - 1.626) | 1.043 (0.964 - 1.129) |  |  |  |
| Q3 | 1.444 (1.038 - 2.01) | 1.35 (1.047 - 1.743) | 1.043 (0.956 - 1.138) | 1.228 (1.109 - 1.359) | 1.118 (0.947 - 1.32) | 1.286 (1.041 - 1.587) | 0.991 (0.789 - 1.246) | 1.337 (0.911 - 1.964) | 1.121 (1.025 - 1.226) |  |  |  |
| Q4 | 1.478 (0.983 - 2.224) | 1.803 (1.333 - 2.439) | 1.039 (0.935 - 1.155) | 1.359 (1.206 - 1.532) | 1.331 (1.095 - 1.617) | 1.262 (0.979 - 1.627) | 1.121 (0.856 - 1.468) | 1.821 (1.159 - 2.861) | 1.313 (1.18 - 1.462) |  |  |  |
| p-value | 0.156 | 0.002 | 0.817 | <0.001 | 0.011 | 0.069 | 0.501 | 0.062 | <0.001 |  |  |  |
| p-for trend | 0.032 | <0.001 | 0.45 | <0.001 | 0.003 | 0.09 | 0.648 | 0.01 | <0.001 |  |  |  |
| Women (n) | 122 | 41 | 2117 | 2410 | 605 | 663 | 632 | 14 | 1599 | 938 | 360 | 111 |
| Q1 | 1 | 1 | 1 | 1 | 1 | 1 | 1 | 1 | 1 | 1 | 1 | 1 |
| Q2 | 0.717 (0.414 - 1.244) | 3.711 (1.453 - 9.476) | 1.193 (1.049 - 1.358) | 1.007 (0.891 - 1.138) | 0.885 (0.699 - 1.122) | 1.155 (0.91 - 1.465) | 0.861 (0.682 - 1.085) | 0.346 (0.063 - 1.891) | 1.02 (0.883 - 1.179) | 1.089 (0.886 - 1.34) | 0.95 (0.692 - 1.303) | 1.132 (0.636 - 2.018) |
| Q3 | 0.75 (0.417 - 1.348) | 2.605 (0.853 - 7.95) | 1.203 (1.043 - 1.387) | 1.144 (1.004 - 1.304) | 0.955 (0.74 - 1.233) | 1.261 (0.978 - 1.626) | 0.882 (0.684 - 1.136) | 0.469 (0.091 - 2.408) | 1.003 (0.853 - 1.178) | 1.131 (0.911 - 1.403) | 1.119  (0.8 - 1.565) | 1.002 (0.538 - 1.869) |
| Q4 | 0.928 (0.487 - 1.768) | 2.628 (0.729 - 9.476) | 1.371 (1.168 - 1.61) | 1.139 (0.98 - 1.323) | 0.809 (0.598 - 1.093) | 1.265 (0.948 - 1.689) | 0.892 (0.667 - 1.193) | 0.355 (0.057 - 2.223) | 1.088 (0.906 - 1.307) | 0.898  (0.7 - 1.153) | 1.114 (0.756 - 1.64) | 0.771 (0.373 - 1.594) |
| p-value | 0.527 | 0.052 | 0.002 | 0.093 | 0.448 | 0.324 | 0.626 | 0.586 | 0.73 | 0.063 | 0.739 | 0.634 |
| p-for trend | 0.905 | 0.275 | <0.001 | 0.033 | 0.269 | 0.1 | 0.506 | 0.334 | 0.441 | 0.378 | 0.433 | 0.401 |
| WC, cm | Ovary | Prostate | Testicular | Renal | Bladder | CNS | Thyroid | Hodgikin | non-Hodgikin | Multiple myeloma | Leukemia | Etc. |
| Total (n) |  |  |  | 620 | 1555 | 417 | 999 | 14 | 792 | 375 | 473 | 3723 |
| Q1 |  |  |  | 1 | 1 | 1 | 1 | 1 | 1 | 1 | 1 | 1 |
| Q2 |  |  |  | 1.301 (0.988 - 1.714) | 0.983 (0.837 - 1.155) | 1.069 (0.788 - 1.451) | 1.080 (0.897 - 1.301) | 0.284 (0.028 - 2.861) | 1.010 (0.807 - 1.265) | 0.785 (0.573 - 1.077) | 0.863 (0.652 - 1.142) | 1.109 (1.004 - 1.224) |
| Q3 |  |  |  | 1.461 (1.104 - 1.932) | 1.003 (0.848 - 1.186) | 1.134 (0.823 - 1.562) | 1.009 (0.825 - 1.234) | 0.930 (0.177 - 4.885) | 1.034 (0.818 - 1.308) | 0.761 (0.545 - 1.062) | 0.871 (0.647 - 1.172) | 1.088 (0.978 - 1.210) |
| Q4 |  |  |  | 1.447 (1.037 - 2.019) | 0.977 (0.796 - 1.199) | 1.354 (0.924 - 1.986) | 1.091 (0.853 - 1.395) | 0.730 (0.095 - 5.603) | 1.225 (0.926 - 1.622) | 0.994 (0.671 - 1.473) | 1.101 (0.772 - 1.570) | 1.228 (1.079 - 1.397) |
| p-value |  |  |  | 0.069 | 0.982 | 0.422 | 0.744 | 0.717 | 0.324 | 0.109 | 0.208 | 0.013 |
| p-for trend |  |  |  | 0.032 | 0.893 | 0.126 | 0.674 | 0.972 | 0.168 | 0.892 | 0.658 | 0.006 |
| Men (n) |  | 4381 | 12 | 438 | 1311 | 221 | 203 | 10 | 512 | 206 | 283 | 2053 |
| Q1 |  | 1 | 1 | 1 | 1 | 1 | 1 | 1 | 1 | 1 | 1 | 1 |
| Q2 |  | 0.998 (0.911 - 1.094) | 1.354 (0.24 - 7.637) | 1.568 (1.156 - 2.126) | 0.969 (0.823 - 1.142) | 1.024 (0.679 - 1.542) | 0.902 (0.592 - 1.375) | 0.83 (0.123 - 5.594) | 0.921 (0.703 - 1.207) | 0.983 (0.634 - 1.524) | 0.884 (0.614 - 1.271) | 1.071 (0.939 - 1.222) |
| Q3 |  | 1.01 (0.915 - 1.116) | 1.481 (0.216 - 10.162) | 1.357 (0.968 - 1.901) | 0.975 (0.813 - 1.169) | 1.042 (0.665 - 1.632) | 0.776 (0.487 - 1.238) | 0.441 (0.036 - 5.483) | 0.979 (0.731 - 1.31) | 0.955 (0.59 - 1.548) | 1.098 (0.746 - 1.617) | 1.163 (1.006 - 1.344) |
| Q4 |  | 0.948 (0.841 - 1.068) | 1.785 (0.18 - 17.704) | 1.561 (1.059 - 2.301) | 0.997 (0.802 - 1.238) | 1.241 (0.735 - 2.097) | 0.819 (0.474 - 1.415) | 1.757 (0.173 - 17.853) | 1.052 (0.745 - 1.486) | 1.692 (0.994 - 2.881) | 1.26 (0.795 - 1.999) | 1.163 (0.977 - 1.386) |
| p-value |  | 0.558 | 0.969 | 0.032 | 0.971 | 0.799 | 0.763 | 0.674 | 0.802 | 0.029 | 0.378 | 0.216 |
| p-for trend |  | 0.458 | 0.63 | 0.099 | 0.988 | 0.438 | 0.404 | 0.712 | 0.687 | 0.059 | 0.228 | 0.057 |
| Women (n) | 221 |  |  | 182 | 244 | 196 | 796 | 4 | 280 | 169 | 190 | 1670 |
| Q1 | 1 |  |  | 1 | 1 | 1 | 1 | 1 | 1 | 1 | 1 | 1 |
| Q2 | 1.242 (0.836 - 1.845) |  |  | 0.877 (0.547 - 1.406) | 1.088 (0.733 - 1.615) | 1.261 (0.823 - 1.932) | 1.098 (0.888 - 1.359) | 0.575  (0 - .) | 0.925 (0.642 - 1.333) | 0.891 (0.584 - 1.361) | 0.783 (0.515 - 1.192) | 1.001 (0.867 - 1.155) |
| Q3 | 1.145 (0.728 - 1.802) |  |  | 1.102 (0.682 - 1.781) | 1.25 (0.824 - 1.896) | 1.281 (0.802 - 2.044) | 1.167 (0.929 - 1.467) | >999  (0 - .) | 1.057 (0.716 - 1.56) | 0.657 (0.399 - 1.084) | 0.83 (0.526 - 1.312) | 1.064 (0.91 - 1.245) |
| Q4 | 1.875 (1.155 - 3.044) |  |  | 1.085 (0.629 - 1.871) | 1.212 (0.754 - 1.95) | 1.298 (0.76 - 2.217) | 1.034 (0.79 - 1.352) | >999  (0 - .) | 1.369 (0.89 - 2.106) | 0.641 (0.362 - 1.134) | 0.798 (0.469 - 1.356) | 1.063 (0.888 - 1.273) |
| p-value | 0.041 |  |  | 0.77 | 0.754 | 0.7 | 0.442 | 0.924 | 0.217 | 0.311 | 0.706 | 0.798 |
| p-for trend | 0.022 |  |  | 0.57 | 0.363 | 0.379 | 0.726 | 0.472 | 0.117 | 0.077 | 0.469 | 0.398 |
| Abbreviations: WC, waist circumference  Adjusted for age, sex, body mass index, alcohol, smoking, exercise, fasting glucose, income, charlson comorbidity index  Sex-specific cancers are labeled to each sex | | | | | | | | | | | | |

| **Supplementary table 4.** Hazard ratios for cancer according to WC quartiles by BMI categories | | | | | | |
| --- | --- | --- | --- | --- | --- | --- |
|  | BMI<18.5 | 18.5≤BMI<23 | 23≤BMI<25 | 25≤BMI<27.5 | 27.5≤BMI<30 | BMI≥30 |
| WC |  |  |  |  |  |  |
| Total |  |  |  |  |  |  |
| Q1 | 1 | 1 | 1 | 1 | 1 | 1 |
| Q2 | 1.076 (0.942 - 1.228) | 1.024 (0.979 - 1.070) | 1.071 (1.011 - 1.135) | 1.014 (0.951 - 1.081) | 1.026 (0.918 - 1.148) | 1.093 (0.898 - 1.331) |
| Q3 | 1.109 (0.969 - 1.268) | 1.047 (1.002 - 1.093) | 1.107 (1.042 - 1.177) | 1.046 (0.980 - 1.116) | 1.038 (0.928 - 1.160) | 1.125 (0.919 - 1.376) |
| Q4 | 1.130 (0.987 - 1.293) | 1.075 (1.029 - 1.123) | 1.153 (1.087 - 1.222) | 1.157 (1.083 - 1.236) | 1.039 (0.928 - 1.163) | 1.089 (0.892 - 1.328) |
| p-value | 0.32 | 0.009 | <0.001 | <0.001 | 0.911 | 0.699 |
| p-for trend | 0.074 | 0.001 | <0.001 | <0.001 | 0.513 | 0.424 |
| Men |  |  |  |  |  |  |
| Q1 | 1 | 1 | 1 | 1 | 1 | 1 |
| Q2 | 1.086 (0.938 - 1.257) | 1.017 (0.969 - 1.068) | 1.078 (1.011 - 1.149) | 0.974 (0.901 - 1.052) | 1.030 (0.894 - 1.186) | 1.137 (0.846 - 1.528) |
| Q3 | 1.076 (0.922 - 1.256) | 1.035 (0.985 - 1.088) | 1.138 (1.065 - 1.217) | 0.999 (0.928 - 1.076) | 1.041 (0.905 - 1.197) | 1.011 (0.736 - 1.390) |
| Q4 | 1.152 (0.987 - 1.343) | 1.079 (1.028 - 1.132) | 1.160 (1.086 - 1.240) | 1.158 (1.079 - 1.243) | 0.982 (0.847 - 1.139) | 1.322 (0.978 - 1.786) |
| p-value | 0.354 | 0.015 | <0.001 | <0.001 | 0.836 | 0.223 |
| p-for trend | 0.101 | 0.002 | <0.001 | <0.001 | 0.859 | 0.133 |
| Women |  |  |  |  |  |  |
| Q1 | 1 | 1 | 1 | 1 | 1 | 1 |
| Q2 | 1.237 (0.952 - 1.607) | 1.049 (0.968 - 1.136) | 1.158 (1.051 - 1.275) | 1.038 (0.935 - 1.153) | 1.037 (0.889 - 1.210) | 0.961 (0.754 - 1.225) |
| Q3 | 1.122 (0.855 - 1.473) | 1.018 (0.939 - 1.104) | 1.125 (1.025 - 1.234) | 1.117 (1.014 - 1.230) | 0.978 (0.836 - 1.144) | 0.933 (0.735 - 1.184) |
| Q4 | 1.240 (0.945 - 1.628) | 1.074 (0.990 - 1.165) | 1.110 (1.006 - 1.224) | 1.188 (1.078 - 1.309) | 1.093 (0.933 - 1.280) | 0.971 (0.766 - 1.232) |
| p-value | 0.349 | 0.328 | 0.018 | 0.003 | 0.531 | 0.953 |
| p-for trend | 0.236 | 0.163 | 0.059 | <0.001 | 0.431 | 0.752 |
| Abbreviations: WC, waist circumference; BMI, body mass index  Adjusted for age, sex, alcohol, smoking, exercise, fasting glucose, income, charlson comorbidity index; sex was excluded from gender analysis | | | | | | |

| **Supplementary table 5-1**. Hazard ratios and confidence intervals for developing cancer according to quartiles of BMI after excluding those who were diagnosed with cancer within 2 years from the baseline health examination (N=239,813) | | | | | | | | |
| --- | --- | --- | --- | --- | --- | --- | --- | --- |
| BMI | Range | Events  (n) | Follow-up duration (person-year) | Incidence rate  (per 1000 person-years) | Crude | Model 1 | Model 2 | Model 3 |
| Total |  |  |  |  |  |  |  |  |
| Q1 (n=58,363) | 15-21.4 | 9042 | 576601.8563 | 15.6815312 | 1 | 1 | 1 | 1 |
| Q2 (n=60,522) | 21.5-23.4 | 9080 | 620026.5517 | 14.64453413 | 0.925 (0.899 - 0.953) | 0.956 (0.928 - 0.984) | 0.913 (0.885 - 0.942) | 0.945 (0.916 - 0.975) |
| Q3 (n=59,753) | 23.5-25.4 | 8893 | 618711.0007 | 14.3734312 | 0.906 (0.88 - 0.933) | 0.961 (0.933 - 0.990) | 0.889 (0.858 - 0.921) | 0.938 (0.905 - 0.971) |
| Q4 (n=61,175) | 25.5-39.8 | 8542 | 638981.8262 | 13.36814233 | 0.841 (0.816 - 0.866) | 0.969 (0.940 - 0.998) | 0.858 (0.822 - 0.895) | 0.916 (0.878 - 0.956) |
| p-value |  |  |  |  | <0.001 | 0.012 | <0.001 | <0.001 |
| p-for trend |  |  |  |  | <0.001 | 0.061 | <0.001 | <0.001 |
| Men |  |  |  |  |  |  |  |  |
| Q1 (n=30,255) | 15-21.2 | 6210 | 284430.3354 | 21.83311422 | 1 | 1 | 1 | 1 |
| Q2 (n=29,953) | 21.3-23.1 | 6011 | 294215.2471 | 20.4306203 | 0.924 (0.892 - 0.958) | 0.94 (0.907 - 0.974) | 0.892 (0.859 - 0.927) | 0.931 (0.896 - 0.968) |
| Q3 (n=30,167) | 23.2-25.0 | 6061 | 300437.0951 | 20.17394023 | 0.909 (0.878 - 0.942) | 0.935 (0.903 - 0.969) | 0.857 (0.821 - 0.894) | 0.918 (0.88 - 0.959) |
| Q4 (n=30,468) | 25.1-39.8 | 6022 | 305312.8104 | 19.72403317 | 0.888 (0.857 - 0.92) | 0.92 (0.888 - 0.953) | 0.802 (0.762 - 0.844) | 0.878 (0.834 - 0.925) |
| p-value |  |  |  |  | <0.001 | <0.001 | <0.001 | <0.001 |
| p-for trend |  |  |  |  | <0.001 | <0.001 | <0.001 | <0.001 |
| Women |  |  |  |  |  |  |  |  |
| Q1 (n=29,253) | 15-21.7 | 2627 | 306739.0007 | 8.564284275 | 1 | 1 | 1 | 1 |
| Q2 (n=29,689) | 21.8-23.7 | 2829 | 317996.2875 | 8.896330276 | 1.033 (0.98 - 1.09) | 1.058 (1.003 - 1.115) | 1.023 (0.967 - 1.082) | 1.03 (0.974 - 1.09) |
| Q3 (n=30,577) | 23.8-25.9 | 2893 | 328685.9466 | 8.801714919 | 1.021 (0.969 - 1.077) | 1.053 (0.998 - 1.11) | 0.994 (0.935 - 1.057) | 1.004 (0.944 - 1.068) |
| Q4 (n=29,451) | 26.0-39.8 | 2904 | 316504.512 | 9.17522465 | 1.065 (1.011 - 1.123) | 1.099 (1.042 - 1.159) | 1.002 (0.932 - 1.078) | 1.014 (0.943 - 1.091) |
| p-value |  |  |  |  | 0.119 | 0.007 | 0.739 | 0.699 |
| p-for trend |  |  |  |  | 0.037 | 0.001 | 0.809 | 0.938 |
| Abbreviation: BMI, body mass index  Model 1: adjusted for age, sex  Model 2: adjusted for model 1 plus waist circumference  Model 3: adjusted for model 2 plus alcohol, smoking, exercise, fasting blood glucose, income, charlson comorbidity index  In sex-stratified analyses, sex was excluded from the covariates | | | | | | | | |

| **Supplementary table 5-2**. Hazard ratios and confidence intervals for developing cancer according to quartiles of WC after excluding those who were diagnosed with cancer within 2 years from the baseline health examination (N=239,813) | | | | | | | | |
| --- | --- | --- | --- | --- | --- | --- | --- | --- |
| WC | Range | Events  (n) | Follow-up duration (person-year) | Incidence rate  (per 1000 person-years) | Crude | Model 1 | Model 2 | Model 6 |
| Total |  |  |  |  |  |  |  |  |
| Q1 (n=60.816) | 33-76 | 7931 | 620383.7071 | 12.78402368 | 1 | 1 | 1 | 1 |
| Q2 (n=53.984) | 77-81 | 7786 | 555314.2231 | 14.02089065 | 1.095 (1.061 - 1.129) | 1.019 (0.988 - 1.051) | 1.050 (1.016 - 1.086) | 1.045 (1.011 - 1.080) |
| Q3 (n=66.015) | 82-87 | 10093 | 677499.1102 | 14.89743654 | 1.164 (1.13 - 1.199) | 1.020 (0.990 - 1.051) | 1.076 (1.039 - 1.114) | 1.068 (1.032 - 1.106) |
| Q4 (n=58.998) | 88-129 | 9747 | 601124.1944 | 16.21461936 | 1.269 (1.232 - 1.307) | 1.055 (1.023 - 1.087) | 1.150 (1.103 - 1.200) | 1.125 (1.079 - 1.174) |
| p-value |  |  |  |  | <0.001 | 0.005 | <0.001 | <0.001 |
| p-for trend |  |  |  |  | <0.001 | 0.001 | <0.001 | <0.001 |
| Men |  |  |  |  |  |  |  |  |
| Q1 (n=31,667) | 33-78 | 6257 | 303443.7071 | 20.61996955 | 1 | 1 | 1 | 1 |
| Q2 (n=28,715) | 79-83 | 5744 | 282617.1417 | 20.32431567 | 0.978 (0.944 - 1.014) | 0.993 (0.958 - 1.029) | 1.046 (1.007 - 1.087) | 1.041 (1.002 - 1.082) |
| Q3 (n=29,476) | 84-88 | 5936 | 292160.3641 | 20.31760885 | 0.976 (0.942 - 1.012) | 0.995 (0.96 - 1.031) | 1.086 (1.041 - 1.133) | 1.077 (1.032 - 1.124) |
| Q4 (n=30,985) | 89-129 | 6367 | 306174.2752 | 20.79534604 | 1 (0.966 - 1.036) | 1.016 (0.981 - 1.052) | 1.169 (1.111 - 1.229) | 1.128 (1.073 - 1.187) |
| p-value |  |  |  |  | 0.358 | 0.57 | <0.001 | <0.001 |
| p-for trend |  |  |  |  | >0.999 | 0.373 | <0.001 | <0.001 |
| Women |  |  |  |  |  |  |  |  |
| Q1 (n=31,323) | 36-75 | 2749 | 332248.3039 | 8.27393238 | 1 | 1 | 1 | 1 |
| Q2 (n=28,674) | 76-80 | 2685 | 307218.7871 | 8.73969989 | 1.054 (0.999 - 1.112) | 1.059 (1.005 - 1.117) | 1.058 (1 - 1.119) | 1.057 (0.999 - 1.118) |
| Q3 (n=27,025) | 81-85 | 2646 | 289263.3785 | 9.147372936 | 1.103 (1.046 - 1.164) | 1.111 (1.053 - 1.172) | 1.108 (1.043 - 1.178) | 1.108 (1.042 - 1.177) |
| Q4 (n=31,948) | 86-129 | 3173 | 341195.2772 | 9.29965979 | 1.123 (1.067 - 1.181) | 1.119 (1.063 - 1.178) | 1.115 (1.04 - 1.196) | 1.109 (1.034 - 1.189) |
| p-value |  |  |  |  | <0.001 | <0.001 | 0.006 | 0.008 |
| p-for trend |  |  |  |  | <0.001 | <0.001 | 0.001 | 0.002 |
| Abbreviation: WC, waist circumference  Model 1: adjusted for age, sex  Model 2: adjusted for model 1 plus BMI  Model 3: adjusted for model 2 plus alcohol, smoking, exercise, fasting blood glucose, income, charlson comorbidity index | | | | | | | | |

| **Supplementary table 6-1**. Hazard ratios and confidence intervals for developing cancer according to quartiles of BMI after excluding those who were diagnosed with cancer within 3 years from the baseline health examination, stratified by smoking history  **Never smoker (N=162725)** | | | | | | | | |
| --- | --- | --- | --- | --- | --- | --- | --- | --- |
| BMI | Range | Events  (n) | Follow-up duration (person-year) | Incidence rate  (per 1000 person-years) | Crude | Model 1 | Model 2 | Model 3 |
| Total |  |  |  |  |  |  |  |  |
| Q1 (n=40500) | 15 - 21.7 | 4134 | 419505.5 | 9.854459596 | 1 | 1 | 1 | 1 |
| Q2 (n=39733) | 21.8 - 23.6 | 4349 | 420938.2 | 10.33168289 | 1.041 (0.997 - 1.086) | 1.059 (1.015 - 1.105) | 1.055 (1.011 - 1.101) | 1.005 (0.960 - 1.052) |
| Q3 (n=41888) | 23.7 - 25.7 | 4607 | 446469.34 | 10.31873768 | 1.037 (0.994 - 1.081) | 1.066 (1.022 - 1.112) | 1.061 (1.017 - 1.107) | 0.978 (0.931 - 1.027) |
| Q4 (n=40604) | 25.8 - 39.8 | 4293 | 435108.99 | 9.86649345 | 0.990 (0.948 - 1.033) | 1.082 (1.037 - 1.130) | 1.073 (1.028 - 1.121) | 0.943 (0.888 - 1.000) |
| *p*-value |  |  |  |  | 0.039 | 0.002 | 0.007 | 0.089 |
| Men |  |  |  |  |  |  |  |  |
| Q1 (n=12147) | 15 - 21.5 | 1832 | 119543.7 | 15.32493975 | 1 | 1 | 1 | 1 |
| Q2 (n=12576) | 21.6 - 23.4 | 1990 | 127713.23 | 15.58178428 | 1.005 (0.943 - 1.071) | 1.026 (0.963 - 1.093) | 1.020 (0.957 - 1.086) | 0.956 (0.893 - 1.023) |
| Q3 (n=12873) | 23.5 - 25.3 | 2085 | 132388.55 | 15.74909613 | 1.011 (0.950-1.077) | 1.044 (0.980 - 1.111) | 1.035 (0.972 - 1.102) | 0.929 (0.862 - 1.001) |
| Q4 (n=12254) | 25.4 - 39.8 | 1975 | 126268.73 | 15.64124388 | 1.004 (0.942 - 1.070) | 1.048 (0.983 - 1.117) | 1.033 (0.969 - 1.102) | 0.871 (0.796 - 0.954) |
| *p*-value |  |  |  |  | 0.988 | 0.467 | 0.704 | 0.027 |
| Women |  |  |  |  |  |  |  |  |
| Q1 (n=28828) | 15 - 21.8 | 2287 | 305667.59 | 7.481983942 | 1 | 1 | 1 | 1 |
| Q2 (n=28093) | 21.9 - 23.8 | 2370 | 303831.75 | 7.800369777 | 1.036 (0.978 - 1.097) | 1.058 (0.999 - 1.120) | 1.056 (0.997 - 1.119) | 1.018 (0.958 - 1.082) |
| Q3 (n=27817) | 23.9 - 25.9 | 2362 | 301481.65 | 7.834639355 | 1.039 (0.981 - 1.101) | 1.069 (1.009 - 1.133) | 1.067 (1.007 - 1.130) | 1.003 (0.938 - 1.072) |
| Q4 (n=28137) | 26.0 - 39.8 | 2482 | 305126.83 | 8.134322373 | 1.079 (1.019 - 1.142) | 1.111 (1.049 - 1.176) | 1.106 (1.045 - 1.171) | 1.002 (0.927 - 1.084) |
| *p*-value |  |  |  |  | 0.075 | 0.004 | 0.007 | 0.926 |
| Abbreviation: BMI, body mass index  Model 1: adjusted for age, sex  Model 2: adjusted for model 1 plus alcohol, exercise, fasting blood glucose, income, charlson comorbidity index  Model 3: adjusted for model 2 plus WC  In sex-stratified analyses, sex was excluded from the covariates. | | | | | | | | |

| **Ever smoker (N=71509)** | | | | | | | | |
| --- | --- | --- | --- | --- | --- | --- | --- | --- |
| BMI | Range | Events  (n) | Follow-up duration (person-year) | Incidence rate  (per 1000 person-years) | Crude | Model 1 | Model 2 | Model 3 |
| Total |  |  |  |  |  |  |  |  |
| Q1 (n=17507) | 15 - 20.9 | 3489 | 165112.67 | 21.13102526 | 1 | 1 | 1 | 1 |
| Q2 (n=18127) | 21.0 - 22.9 | 3523 | 179393.53 | 19.63838941 | 0.911 (0.870 - 0.955) | 0.921 (0.879 - 0.965) | 0.925 (0.882 - 0.969) | 0.891 (0.847 - 0.937) |
| Q3 (n=17665) | 23.0 - 24.8 | 3483 | 177038.26 | 19.67371347 | 0.908 (0.867 - 0.952) | 0.927 (0.885 - 0.972) | 0.936 (0.893 - 0.982) | 0.879 (0.830 - 0.930) |
| Q4 (n=18210) | 24.9 - 39.6 | 3500 | 183889.77 | 19.03314143 | 0.876 (0.836 - 0.918) | 0.907 (0.865 - 0.950) | 0.915 (0.873 - 0.960) | 0.830 (0.774 - 0.889) |
| p-value |  |  |  |  | <0.001 | <0.001 | 0.001 | <0.001 |
| Men |  |  |  |  |  |  |  |  |
| Q1 (n=17224) | 15 - 21 | 3542 | 162194.05 | 21.83803906 | 1 | 1 | 1 | 1 |
| Q2 (n=16381) | 21.1 - 22.9 | 3236 | 161859.91 | 19.9925973 | 0.897 (0.856 - 0.941) | 0.914 (0.871 - 0.959) | 0.917 (0.874 - 0.962) | 0.885 (0.841 - 0.932) |
| Q3 (n=16821) | 23.0 - 24.8 | 3377 | 168286.09 | 20.06701802 | 0.896 (0.855 - 0.940) | 0.923 (0.880 - 0.967) | 0.930 (0.887 - 0.975) | 0.876 (0.827 - 0.928) |
| Q4 (n=17081) | 24.9 - 39.5 | 3367 | 172058.39 | 19.5689382 | 0.871 (0.831 - 0.913) | 0.903 (0.862 - 0.947) | 0.909 (0.867 - 0.954) | 0.828 (0.772 - 0.888) |
| p-value |  |  |  |  | <0.001 | <0.001 | <0.001 | <0.001 |
| Women |  |  |  |  |  |  |  |  |
| Q1 (n=1016) | 15 - 20.6 | 107 | 9995.85 | 10.70444234 | 1 | 1 | 1 | 1 |
| Q2  (n=981) | 20.7 - 22.8 | 123 | 10105.84 | 12.17118023 | 1.120 (0.864 - 1.451) | 1.141 (0.879 - 1.480) | 1.143 (0.880 - 1.484) | 1.115 (0.845 - 1.472) |
| Q3  (n=992) | 22.9 - 25.1 | 120 | 10310.66 | 11.63844022 | 1.067 (0.822 - 1.384) | 1.093 (0.841 - 1.422) | 1.096 (0.842 - 1.427) | 1.051 (0.771 - 1.431) |
| Q4 (n=1013) | 25.2 - 39.6 | 123 | 10623.45 | 11.57815964 | 1.058 (0.816 - 1.371) | 1.090 (0.838 - 1.416) | 1.099 (0.843 - 1.433) | 1.025 (0.704 - 1.493) |
| p-value |  |  |  |  | 0.865 | 0.798 | 0.789 | 0.858 |
| Abbreviation: BMI, body mass index  Model 1: adjusted for age, sex  Model 2: adjusted for model 1 plus alcohol, exercise, fasting blood glucose, income, charlson comorbidity index  Model 3: adjusted for model 2 plus WC  In sex-stratified analyses, sex was excluded from the covariates. | | | | | | | | |

| **Supplementary table 6-2**. Hazard ratios and confidence intervals for developing cancer according to quartiles of WC after excluding those who were diagnosed with cancer within 3 years from the baseline health examination, stratified by smoking history  **Never smoker (N=162725)** | | | | | | | | |
| --- | --- | --- | --- | --- | --- | --- | --- | --- |
| WC | Range | Events  (n) | Follow-up duration (person-year) | Incidence rate  (per 1000 person-years) | Crude | Model 1 | Model 2 | Model 3 |
| Total |  |  |  |  |  |  |  |  |
| Q1 (n=44102) | 34 - 76 | 3977 | 466286.92 | 8.529083338 | 1 | 1 | 1 | 1 |
| Q2 (n=37473) | 77 - 81 | 3930 | 398516.57 | 9.861572381 | 1.154 (1.105 - 1.206) | 1.091 (1.044 - 1.140) | 1.087 (1.040 - 1.136) | 1.094 (1.044 - 1.146) |
| Q3 (n=37753) | 82 - 86 | 4235 | 399950.9 | 10.58879978 | 1.242 (1.189 - 1.297) | 1.115 (1.068 - 1.165) | 1.110 (1.062 - 1.159) | 1.122 (1.067 - 1.179) |
| Q4 (n=43397) | 87 - 129 | 5241 | 457267.62 | 11.46155943 | 1.347 (1.293 - 1.404) | 1.151 (1.104 - 1.201) | 1.141 (1.094 - 1.190) | 1.162 (1.097 - 1.230) |
| *p*-value |  |  |  |  | <0.001 | <0.001 | <0.001 | <0.001 |
| Men |  |  |  |  |  |  |  |  |
| Q1 (n=12038) | 34 - 78 | 1753 | 120412.35 | 14.55830735 | 1 | 1 | 1 | 1 |
| Q2 (n=11895) | 79 - 83 | 1870 | 120906.23 | 15.46653138 | 1.056 (0.989 - 1.127) | 1.072 (1.004 - 1.144) | 1.066 (0.999 - 1.138) | 1.094 (1.021 - 1.173) |
| Q3 (n=12634) | 84 - 88 | 2033 | 129446.6 | 15.70531787 | 1.070 (1.004 - 1.141) | 1.094 (1.026 - 1.167) | 1.085 (1.018 - 1.157) | 1.135 (1.053 - 1.223) |
| Q4 (n=13283) | 89 - 129 | 2226 | 135149.02 | 16.47070767 | 1.126 (1.057 - 1.198) | 1.146 (1.076 - 1.220) | 1.128 (1.059 - 1.202) | 1.213 (1.110 - 1.325) |
| *p*-value |  |  |  |  | 0.003 | <0.001 | 0.002 | <0.001 |
| Women |  |  |  |  |  |  |  |  |
| Q1 (n=29501) | 36 - 75 | 2264 | 316085.42 | 7.162620788 | 1 | 1 | 1 | 1 |
| Q2 (n=27233) | 76 - 80 | 2285 | 294325.4 | 7.763516163 | 1.081 (1.020 - 1.146) | 1.086 (1.025 - 1.151) | 1.084 (1.023 - 1.149) | 1.077 (1.013 - 1.145) |
| Q3 (n=25737) | 81 - 85 | 2249 | 278053.44 | 8.088373228 | 1.127 (1.063 - 1.194) | 1.133 (1.069 - 1.201) | 1.131 (1.067 - 1.199) | 1.119 (1.048 - 1.196) |
| Q4 (n=30404) | 86 - 129 | 2703 | 327643.55 | 8.249819049 | 1.150 (1.088 - 1.216) | 1.145 (1.083 - 1.211) | 1.140 (1.077 - 1.206) | 1.121 (1.039 - 1.210) |
| *p*-value |  |  |  |  | <0.001 | <0.001 | <0.001 | 0.007 |
| Abbreviation: BMI, body mass index  Model 1: adjusted for age, sex  Model 2: adjusted for model 1 plus alcohol, exercise, fasting blood glucose, income, charlson comorbidity index  Model 3: adjusted for model 2 plus BMI  In sex-stratified analyses, sex was excluded from the covariates. | | | | | | | | |

| **Ever smoker (N=71509)** | | | | | | | | |
| --- | --- | --- | --- | --- | --- | --- | --- | --- |
| WC | Range | Events  (n) | Follow-up duration (person-year) | Incidence rate  (per 1000 person-years) | Crude | Model 1 | Model 2 | Model 3 |
| Total |  |  |  |  |  |  |  |  |
| Q1 (n=17714) | 33 - 77 | 3388 | 170530.23 | 19.8674452 | 1 | 1 | 1 | 1 |
| Q2 (n=16617) | 78 - 82 | 3236 | 164204.96 | 19.70707828 | 0.981 (0.935 - 1.030) | 0.976 (0.930 - 1.024) | 0.977 (0.931 - 1.026) | 1.014 (0.963 - 1.068) |
| Q3 (n=19725) | 83 - 88 | 3883 | 196695.67 | 19.74115648 | 0.980 (0.935 - 1.026) | 0.971 (0.927 - 1.017) | 0.975 (0.931 - 1.021) | 1.041 (0.984 - 1.100) |
| Q4 (n=17453) | 89 - 129 | 3488 | 174003.36 | 20.04558992 | 0.995 (0.949 - 1.043) | 0.983 (0.938 - 1.031) | 0.984 (0.938 - 1.032) | 1.094 (1.021 - 1.173) |
| *p*-value |  |  |  |  | 0.783 | 0.463 | 0.502 | 0.011 |
| Men |  |  |  |  |  |  |  |  |
| Q1 (n=16037) | 33 - 77 | 3192 | 153514.25 | 20.79285799 | 1 | 1 | 1 | 1 |
| Q2 (n=18689) | 78 - 83 | 3732 | 184474.5 | 20.23043835 | 0.960 (0.916 - 1.007) | 0.977 (0.932 - 1.024) | 0.976 (0.931 - 1.023) | 1.017 (0.967 - 1.071) |
| Q3 (n=15983) | 84 - 88 | 3194 | 159226.9 | 20.05942463 | 0.949 (0.903 - 0.996) | 0.969 (0.922 - 1.017) | 0.971 (0.924 - 1.020) | 1.043 (0.983 - 1.106) |
| Q4 (n=16798) | 89 - 125 | 3404 | 167182.78 | 20.36094866 | 0.963 (0.918 - 1.011) | 0.981 (0.935 - 1.029) | 0.977 (0.930 - 1.025) | 1.093 (1.018 - 1.173) |
| *p*-value |  |  |  |  | 0.171 | 0.624 | 0.64 | 0.066 |
| Women |  |  |  |  |  |  |  |  |
| Q1  (n=974) | 53 - 73 | 110 | 9790.02 | 11.2359321 | 1 | 1 | 1 | 1 |
| Q2 (n=1049) | 74 - 79 | 122 | 10786.28 | 11.31066503 | 0.997 (0.771 - 1.291) | 1.004 (0.775 - 1.299) | 1.012 (0.781 - 1.310) | 1.022 (0.778 - 1.342) |
| Q3 (n=1004) | 80 - 85 | 117 | 10371.84 | 11.28054424 | 0.994 (0.766 - 1.289) | 1.002 (0.772 - 1.300) | 1.015 (0.781 - 1.320) | 1.033 (0.762 - 1.400) |
| Q4  (n=975) | 86 - 129 | 124 | 10087.66 | 12.29224617 | 1.084 (0.838 - 1.401) | 1.093 (0.846 - 1.414) | 1.103 (0.852 - 1.430) | 1.136 (0.787 - 1.638) |
| *p*-value |  |  |  |  | 0.887 | 0.874 | 0.865 | 0.891 |
| Abbreviation: BMI, body mass index  Model 1: adjusted for age, sex  Model 2: adjusted for model 1 plus alcohol, exercise, fasting blood glucose, income, charlson comorbidity index  Model 3: adjusted for model 2 plus BMI  In sex-stratified analyses, sex was excluded from the covariates | | | | | | | | |

**Supplementary Figure legends**

**Supplementary Figure 1.** Enrollment of study participants and exclusion criteria

**Supplementary Figure 2.** Hazard ratios and confidence intervals for cancer incidence of specific cancer types using Cox proportional hazard models. 2-1). BMI, 2-2). WC were categorized into quartiles. Models were adjusted for age, sex, alcohol, smoking, exercise, fasting blood glucose, income, Charlson comorbidity index, and mutually adjusted for WC and BMI

**Supplementary Figure 3**. Heatmap illustrating cancer risk according to BMI category and WC quartiles
